# Snowflake-inspired and blink-driven flexible piezoelectric contact lenses for effective corneal injury repair

**DOI:** 10.1038/s41467-023-39315-6

**Published:** 2023-06-17

**Authors:** Guang Yao, Xiaoyi Mo, Shanshan Liu, Qian Wang, Maowen Xie, Wenhao Lou, Shiyan Chen, Taisong Pan, Ke Chen, Dezhong Yao, Yuan Lin

**Affiliations:** 1 School of Materials and Energy, University of Electronic Science and Technology of China, Chengdu, 610054 Sichuan China; 2 State Key Laboratory of Electronic Thin films and Integrated Devices, University of Electronic Science and Technology of China, Chengdu, 610054 Sichuan China; 3 Shenzhen Institute for Advanced Study, University of Electronic Science and Technology of China, Shenzhen, 518110 China; 4 MOE Key Laboratory for Neuroinformation, The Clinical Hospital of Chengdu Brain Sciences Institute, University of Electronic Science and Technology of China, Chengdu, 610054 Sichuan China; 5 Department of Ophthalmology, Sichuan Academy of Medical Sciences and Sichuan Provincial People’s Hospital, Medical School, University of Electronic Science and Technology of China, Chengdu, 610054 Sichuan China; 6 Medico-Engineering Cooperation on Applied Medicine Research Center, University of Electronic Science and Technology of China, Chengdu, 610054 Sichuan China

**Keywords:** Electronic devices, Electrical and electronic engineering, Mechanical engineering, Corneal diseases, Disease prevention

## Abstract

The cornea is a tissue susceptible to various injuries and traumas with a complicated cascade repair process, in which conserving its integrity and clarity is critical to restoring visual function. Enhancing the endogenous electric field is recognized as an effective method of accelerating corneal injury repair. However, current equipment limitations and implementation complexities hinder its widespread adoption. Here, we propose a snowflake-inspired, blink-driven flexible piezoelectric contact lens that can convert mechanical blink motions into a unidirectional pulsed electric field for direct application to moderate corneal injury repair. The device is validated on mouse and rabbit models with different relative corneal alkali burn ratios to modulate the microenvironment, alleviate stromal fibrosis, promote orderly epithelial arrangement and differentiation, and restore corneal clarity. Within an 8-day intervention, the corneal clarity of mice and rabbits improves by more than 50%, and the repair rate of mouse and rabbit corneas increases by over 52%. Mechanistically, the device intervention is advantageous in blocking growth factors’ signaling pathways specifically involved in stromal fibrosis whilst preserving and harnessing the signaling pathways required for indispensable epithelial metabolism. This work put forward an efficient and orderly corneal therapeutic technology utilizing artificial endogenous-strengthened signals generated by spontaneous body activities.

## Abstract (web summary)

The cornea is susceptible to various injuries with a complicated repair process. Here, the authors propose a snowflake-inspired, blink-driven flexible piezoelectric contact lens for corneal injury repair.

## Introduction

The eye is a natural open window for the interaction between the human and the outside world, and the cornea is the first barrier to defending against external invasion. The ocular surface location makes the cornea susceptible to infection and injury, and corneal injury is a common ophthalmological disease^1,2^. Mild corneal injuries can be completely self-restituted. In contrast, moderate or severe corneal injuries will cause irreversible damage, among which corneal alkali burn is a leading cause (7.0–9.9%) of visual dysfunction and blindness^3–5^. The treatment purpose of corneal injury is not only the physical wound closure, more importantly, the orderly tissue arrangement, clarity recovery, and vision function restoration^6,7^. The primary clinical therapeutic modalities for corneal injuries include drug therapy, corneal transplantation, and stem cell therapy. Although some drug treatments can reduce stromal inflammation to achieve high corneal clarity, promoting orderly tissue arrangement and avoiding side effects is challenging^8^. Corneal transplantation is an effective method for treating refractory or severely defective corneal diseases. However, despite the high success rate, increasing demands for donor corneas and graft rejection of severe immune responses are the primary reasons for developing alternatives to this procedure^9,10^. Cell therapy can avoid rejection reactions and promote corneal repair well, but it is still in the preclinical stage and prolongs the treatment cycle^3,11,12^. Therefore, how to repair corneal injury conveniently, orderly, and efficiently is a challenge facing clinical medicine.

Endogenous electric field (EF) is a spontaneous EF formed after corneal injury, promoting orderly structural arrangement and visual function restoration^13,14^. However, the capacity of endogenous EF alone to repair the irreversible corneal injury is insufficient, and untimely intervention will cause limbal stem cells deficiency (LSCD), accompanied by corneal conjunctivalization and vascularization^15^. Augmentation of the endogenous EF by applying an external EF has been proven to accelerate orderly corneal repair^16,17^. Nonetheless, equipment limitations and implementation complexities hinder its widespread adoption^18,19^. Furthermore, during the repair process of corneal injury, the friction between the eyelid and the cornea during blink (pressure ~ 4 kPa) will cause secondary damage, considering that humans naturally blink around 10,000 times a day^20,21^. Also, long-term treatment with eye closure will cause problems such as hypoxia and edema, resulting in further condition deterioration^22^. Bandage contact lens, as a clinical physical therapy method, has been widely used to avoid secondary damage caused by eyelid friction after corneal defects, such as laser injury and alkali burn^23,24^. However, its weak repair capability could not accelerate the inherent repair process or alleviate the stromal inflammatory reaction. More recently, sophisticated contact lenses powered by various energy supply modalities (battery, capacitor, wireless transmission, etc.) provided an advanced platform for eye health management, which has exhibited great potential for diagnosis and therapeutics of eye diseases^25–28^. However, issues such as chemical leakage, heat generation, and wireless electromagnetic crosstalk need critical investigation, assessment, and circumventing^29–31^. Hence, finding a safe and adequate power supply to drive contact lenses for corneal repair is the key to solving this problem. Recent studies revealed that self-powered bioelectronics could directly establish a conversion bridge between electrical signals and biomechanical motions, thus providing a promising self-responsive diagnosis and therapeutic platform^32,33^.

Here, we proposed a snowflake-inspired and blink-driven piezoelectric contact lens (BPCL), which can avoid the secondary corneal damage from eyelid friction and convert the daily blink motions into a pulsed EF to strengthen the endogenous EF. The flexible BPCL comprised a piezoelectric electret film with a fractal structure, a miniaturized rectifier, and a pair of transparent and extensible stimulating electrodes. We implemented device verification on mouse and rabbit models with moderate corneal injury to systematically investigate the repair effect on different relative alkali burn ratios. Comprehensive characterization showed that device intervention avoided arrangement disorders of corneal tissue in both models. The mice’s corneal clarity, repair rate, and epithelial thickness improved by more than 53%, 159%, and 36%, respectively. The corneal clarity and repair rate of rabbits increased by over 50% and 52%, respectively. Mechanistically, the BPCL intervention could regulate the dual role of related growth factors to manipulate the corneal microenvironment, alleviate stromal fibrosis, promote the orderly migration and regular arrangement of epithelial cells, and restore corneal clarity. This work provided an orderly and efficient corneal injury repair strategy in a self-responsive, battery-free paradigm, directly correlating daily body activities to tissue repair.

## Results

### Design and characterization of the BPCL

**Fig. 1:**
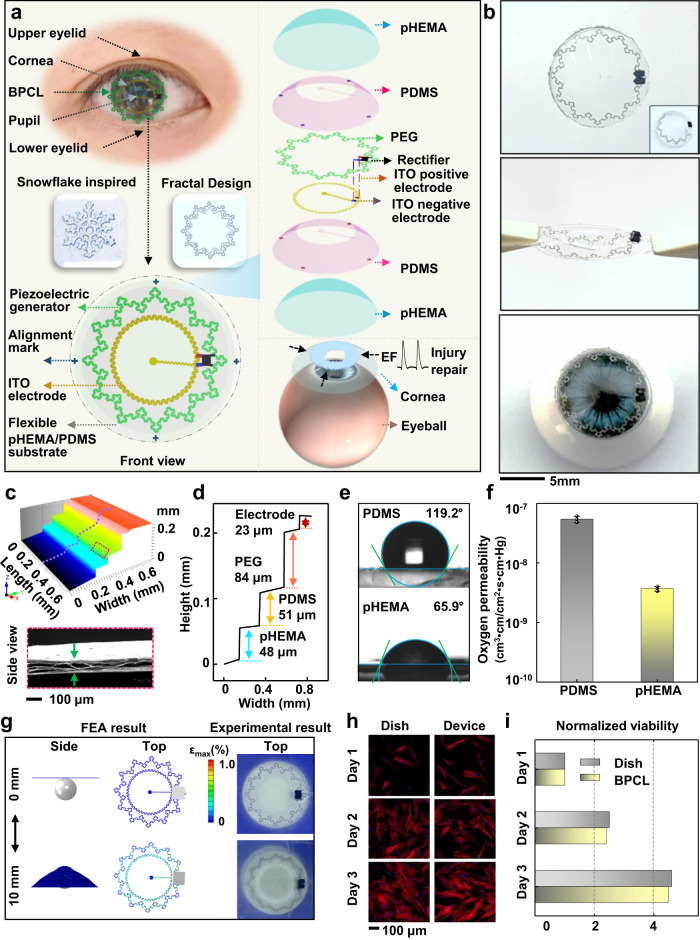
Design and characterization of the BPCL. **a** Schematics of the overall snowflake-inspired BPCL structure (left) and exploded illustration of the device components, essential materials, multilayer structures, and repair mechanism (right). **b** Optical images of an initial BPCL (top) being stretched and twisted (middle) and being worn on an eyeball (bottom). **c** A three-dimensional microscope image of the multilayers and enlarged view of piezoelectric electret film with a porous loose structure (a scanning electron microscope image). **d** The height profile along the purple line in (**c**) shows the height of multilayers. **e** Characterization of the hydrophobic PDMS and the hydrophilic pHEMA encapsulation layers. **f** Oxygen permeability characterization of encapsulation layers, *n* = 3 independent samples. All data in (**f**) are presented as means ± SD. **g** FEA and experimental results of the BPCL under different pressing heights. **h** Fluorescence images of stained fibroblasts cultured on a regular cell culture dish and the encapsulation layer. **i** Comparison of normalized cell viability for three days showing excellent biocompatibility of the BPCL, *n* = 3 independent samples. BPCL blink-driven piezoelectric contact lens, PEG polypropylene electret film generator, PDMS polydimethylsiloxane, ITO indium tin oxide, pHEMA poly 2-hydroxyethyl methacrylate, EF electric field, FEA finite element analysis.

The BPCL-based corneal injury repair system was designed following the principle depicted in Fig. 1a. As shown in Fig. 1a (left), the snowflake-inspired BPCL was built to resemble a cosmetic-colored contact lens to ensure the pupil was not blocked and minimize the impact on intrinsic vision. The BPCL consisted of three modules: a fractal star-polygon piezoelectric polypropylene electret film generator (PEG) with aluminum (Al) electrodes for electric pulse generation, a plate-like concentric circular indium tin oxide (ITO)/ polyethylene terephthalate (PET) film electrodes providing a spatially distributed EF, and a micro rectifier as the intermediate connection rendering the EF directionality. As the exploded illustration was shown in Fig. 1a (right), a pair of serpentine-geometry concentric circular ITO electrodes was deposited 0.3 mm apart from the PEG component and connected to the PEG through the micro rectifier. To avoid potential erosion and ensure wearing comfort in the physiological environment, the functional components were successively encapsulated by patterned polydimethylsiloxane (PDMS) and poly 2-hydroxyethyl methacrylate (pHEMA)^34–36^. The pressure between the eyelid and cornea during blink acted as the sole source to drive the BPCL and generate pulsed voltage signals, which in response stimulated the corneal injury efficient repair through orderly epithelial arrangement and stromal fibrosis alleviation. Fabrication procedures of the BPCL were displayed in Supplementary Fig. 1, including femtosecond laser patterning, template and substrate preparation, transfer printing, integration, and package. Partially enlarged block elements and related parametric characterization of the BPCL were shown in Supplementary Fig. 2a and b. The overall dimensions of the BPCL in the initial state are ~14 × 3.5 × 0.25 (Φ × H × T) mm^3^ (Fig. 1b, top). The fractal structure and serpentine geometry can effectively promote structural robustness, optimize overall modulus, and minimize flexibility restrictions. Therefore, the BPCL could be subjected to considerable deformation, such as stretching and twisting (Fig. 1b, middle). Figure 1b (bottom) demonstrated the experimental wearing mode for BPCL, indicating the BPCL could be seamlessly attached to the eyeball (Supplementary Movie 1).

We employed a three-dimensional microscope to detect the height information of the multi-layered components (Fig. 1c). It is worth noting that the piezoelectric electret film had a porous sectional structure, and abundant air voids were distributed inside the polypropylene architecture, altering surface charge distribution during deformation^37,38^. The cross-sectional height profile was intercepted along a scanning line to quantify the multilayer geometry (Fig. 1d, Supplementary Fig. 2c and d). The thickness of the pHEMA, the PDMS, the PEG, and the ITO/PET layer were 48, 51, 84, and 23 μm, respectively. The hydrophobicity of the encapsulation layers composed of PDMS and pHEMA was shown in Fig. 1e. The contact angles of the PDMS and pHEMA films were 119.2° and 65.9°, confirming their hydrophobic and hydrophilic properties, respectively. Furthermore, the values of the oxygen permeability coefficient were 5.42 × 10^−8^ and 0.37 × 10^−8^ cm^3^•cm/cm^2^•s•cm•Hg for PDMS and pHEMA^34,35^, respectively (Fig. 1f and Supplementary Fig. 3). These water- and oxygen-permeable properties were necessary for long-term stable operation in a biological environment and avoided adverse effects on the eye microenvironment. The mechanical robustness of the flexible BPCL was verified through a pressing system using a spherical plastic ball with a diameter of 12 mm, where the dendrite number (*D*_*n*_) of the star-polygon PEG was set as 12 (Fig. 1g). Finite element analysis (FEA) results of the BPCL under a series of pressing heights consistently indicated that the tensile strain was evenly distributed on the fractal structure and serpentine lines. Both FEA and experimental results displayed similar deformation behaviors. The strain (≤1.0%) sustained by the BPCL was consistently smaller than the failure strain (5%) even when the BPCL was pressed up to 10 mm, exhibiting its excellent stretchability (Supplementary Fig. 4). In addition, the BPCL was also repeatedly pressed from 0 to 10 mm without any structural damage, which further demonstrated the outstanding mechanical robustness of the BPCL. To ensure in vitro and in vivo biocompatibility of the encapsulated device, mouse fibroblast 3T3 cells were first cultured on the packaged BPCL surface and in a reference culture dish for three days to observe and compare the cell attachment, proliferation, and morphology. Cells in both media exhibited similar densities and equivalent morphologies. The fluorescent staining revealed that the cells in both groups could spread and form intact cytoarchitecture (Fig. 1h). The relative viability of cells on encapsulation material was exceeded 98% within three days, comparable to that of the cells cultured in the dish (Fig. 1i). In addition, during in vivo wearing process of rabbits for two weeks, the corneal optical images and the hematoxylin-eosin (H&E) staining result indicated that the BPCL was associated without corneal tissue injury or affecting regular daily activity (Supplementary Fig. 5 and Supplementary Movie 2). These results confirmed that the encapsulated BPCL is non-cytotoxic, biocompatible, and safe.

### Working principle and performance optimization of the BPCL

**Fig. 2:**
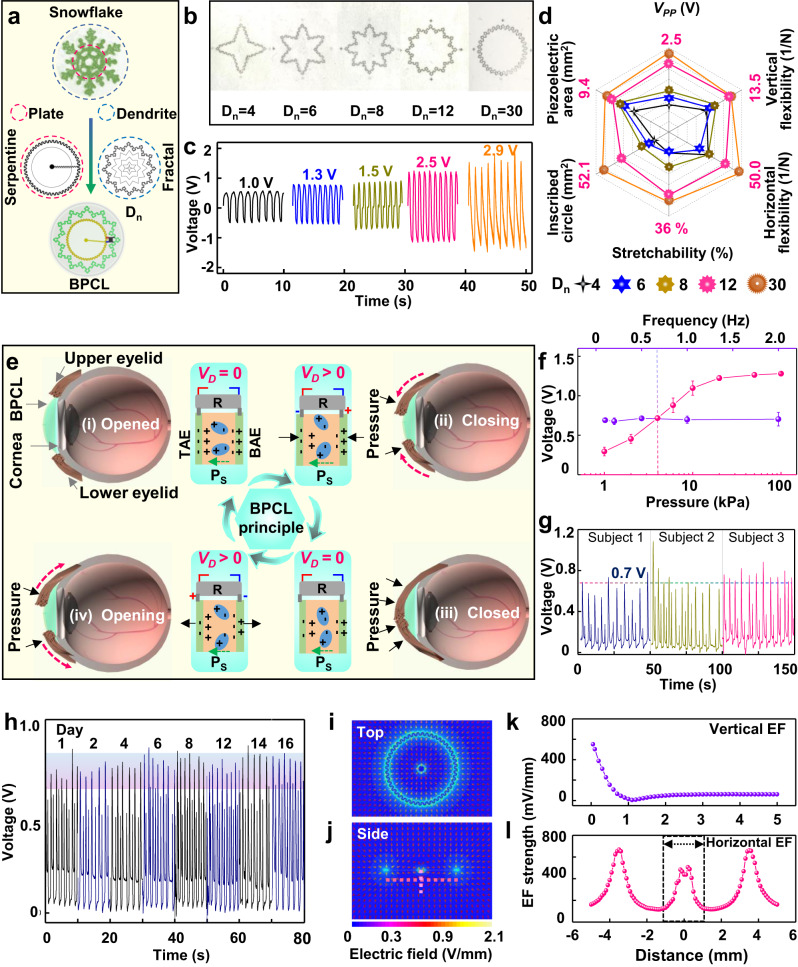
Structural optimization, working principle, and performance of the BPCL. **a** The snowflake-inspired BPCL consisting of inner plate and outer fractal dendrite exhibits self-similarity with the natural snowflake. **b** The fractal star-polygon structure with a different *D*_*n*_. **c** Voltage output comparison of PEGs with a different *D*_*n*_ (4, 6, 8, 12, and 30) at a frequency of 1 Hz. **d** Radar chart of different PEGs for performance comparison. **e** Schematics of the working principle of the BPCL under different eye blink stages (i-iv), R represents the rectifier. **f** *V*_*D*_ recorded from the BPCL under different pressure and frequencies, *n* = 3 independent samples. **g** *V*_*D*_ recorded during blink in vivo. **h** Long-term stability test of the BPCL. **i** Top-view and **j** side-view AMFES simulated EF distribution inside a cornea. **k** Vertical and **l** Horizontal EF strength as a function of the depth and width extrapolated from Fig. 2j. The corneal injury area was marked within the dashed box. All data in (**f**) are presented as means ± SD. BPCL blink-driven piezoelectric contact lens, *D*_*n*_ dendrite number, *V*_*pp*_ peak-to-peak voltage, TAE top aluminum electrode, BAE bottom aluminum electrode, *V*_*D*_ the voltage difference between the ITO electrodes, EF electric field.

The BPCL exhibited self-similarity with natural snowflakes with an inner plate and outer dendrites, which radial-gradient structure radially decreased in porosity from the plate to dendrites (Fig. 2a). The fractal star-polygon structure of the PEG could promote the power generation density and component sensitivity due to the improvement of the piezoelectric area^39–41^ (Supplementary Fig. 6). The open-circuit peak-to-peak voltage of the PEG was defined as *V*_*pp*_. *D*_*n*_ (the number of dendrites) was designed to be 4, 6, 8, 12, and 30 (Fig. 2b), and the corresponding *V*_*pp*_ were 1.0, 1.3, 1.5, 2.5, and 2.9V, respectively (Fig. 2c). Moreover, compared with other geometric parameters (*D*_*n*_ = 4, 6, and 8), comprehensive tests notably indicated that the PEGs (*D*_*n*_ = 12 and *D*_*n*_ = 30) had optimized overall performance under the evaluation criteria of the piezoelectric area, inscribed circle area, voltage output in plane condition, stretchability, and mechanical properties (Fig. 2d, Supplementary Figs. 6, 7, and 8). Considering the voltage output under the actual application scenario (Supplementary Fig. 9), the PEG (*D*_*n*_ = 12) was chosen for further BPCL integration and animal studies.

The polypropylene electret film has a high piezoelectric coefficient (d_33_~145 pC/N) and similar electromechanical behavior to traditional organic piezoelectric materials^42–44^. After polarization, several positive/negative electric charge pairs accumulated on these air voids’ inner top/bottom surfaces (Fig. 1c), forming macroscopic ‘quasi-dipoles’^37,38^. These ‘quasi-dipoles’ generated a strong internal EF and induced opposite polarity charges within the surface electrodes. When the eye was under the daily blink process, the corresponding motion cycle of the PEG layers in the BPCL was illustrated in Fig. 2e (i)-(iv). As the eye was fully opened, the stored charge pairs and the opposite charges induced in the top Al electrode (TAE) and bottom Al electrode (BAE) were in electrostatic equilibrium. There was no voltage difference (*V*_*D*_) between the ITO electrodes connected to the TAE and BAE via the micro rectifier (*V*_*D* _= 0, stage i). The following eye-closing phase pushed the two Al electrodes closer together. The total thickness of the PEG layer was reduced under the pressure of the upper and lower eyelids, which lowered the polarization intensity of the macroscopic dipoles, leading to a negative electric potential in the TAE due to superfluous polarization-induced electrons (stage ii). When the eye was fully closed, the thickness of the PEG decreased to a minimum under eyelid pressure, where the system established a new electrostatic equilibrium (*V*_*D* _= 0, stage iii). The PEG will gradually return to the inherently elastic state following eye-opening (stage iv). The total thickness of the air layers increased, resulting in polarization-induced electrons insufficiency and electric potential rising of the TAE until the eye reached the originally opened stage (i) again. It is worth noting that the voltage polarity at the concentric circular ITO was unidirectionally fixed (*V*_*D* _> 0, outside positive and inside negative) due to the presence of the micro rectifier.

The voltage output performance was monitored first under different test parameters induced by a computer-controlled shaker Fig. 2f (Supplementary Fig. 10a). The output voltage increased as the pressure gradually increased from 1 to 20 kPa and then remained constant (~ 1.3V), while the voltage did not change with various frequencies (0.2–2 Hz, 4 kPa) (Supplementary Fig. 10b and c). The in vivo voltage output during blink in the human-worn state was then monitored. The average voltage output of the three subjects was ~0.7 V (Fig. 2g, Supplementary Movie 3), similar to the output under the pressure of 4 kPa. This comparison confirmed that the BPCL could efficaciously convert spontaneous daily eye blinking movements into steady electric pulses. The long-term stability test of the BPCL showed good device integrity without any observable structural defects and performance degradation after 16 days of operation (Fig. 2h, Supplementary Fig. 10d, Supplementary Fig. 11, Supplementary Movie 4, and Supplementary Movie 5). The almost unchanged voltage amplitude confirmed the excellent stability and robust durability of the BPCL. Ansys Maxwell finite element solver (AMFES) was utilized to evaluate the effectiveness of EF penetration and estimate the EF strength at the corneal injury site. AMFES simulation result indicated that the concentric circular electrodes could construct a uniform EF within their covered corneal area (Fig. 2i, j). Vertical (0–5 mm) and horizontal (−5–5 mm) EF strengths at the corneal injury site extracted from Fig. 2j were shown in Fig. 2k, l, respectively. The vertical EF strength rapidly attenuated within the first 1 mm into the tissue and gradually decayed to a stable value of ∼100 mV/mm at 5 mm deep inside (Fig. 2k). The horizontal EF strength of the injury site at the 200 μm depth was in the approximate range of 120 mV/mm to 500 mV/mm (Fig. 2l). The AMFES simulation evidenced that the BPCL could provide stable and effective EF stimulations into the corneal tissue when covered around the cornea^13,14,45^. Furthermore, since the thickness of the encapsulation layer was only ∼50 μm, it did not introduce any significant impacts on the EF intensity at the corneal injury site. In general, on a limited integration area without affecting intrinsic vision, the snowflake-inspired BPCL based on the fractal polypropylene film with a high piezoelectric coefficient and unidirectional EF generation circuit could render effective intervention for corneal injury repair.

### Corneal repair of mice and rabbits by the BPCL intervention

**Fig. 3:**
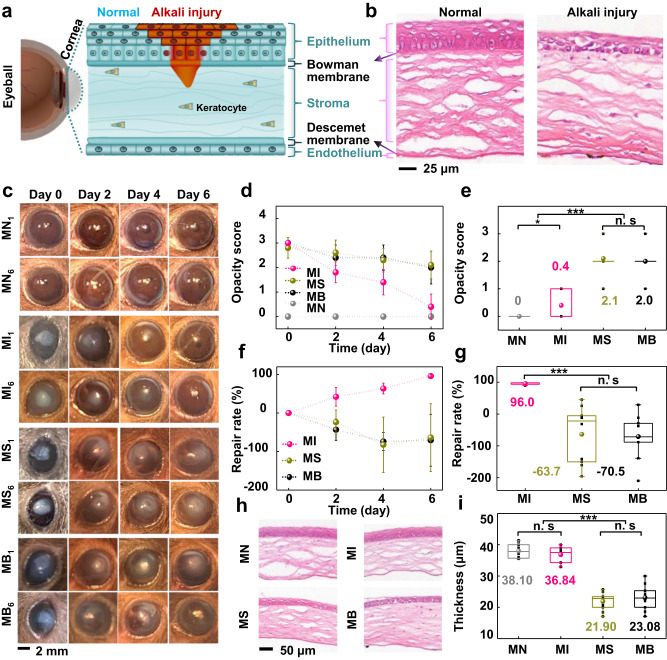
Mice corneal alkali burn repair during the BPCL intervention. **a** The anatomical detail of the injury cornea with the alkali burn area highlighted in red. **b** H&E staining images of the normal cornea and initial alkali burn cornea. **c** A series of images on the alkali burn cornea over time of the MN, MI, MS, and MB groups (*n* = 10) (Male: *n* = 1–5; Female: *n* = 6–10). **d** Corneal opacity score over time in different mouse groups. **e** Comparison of corneal opacity for different mouse groups on day 6. *P* = 0.03359 for MN and MI, *P* < 3.5 × 10^-4^ for MN/MI and MS/MB. **f** Corneal repair rate over time of the injury area. **g** Comparison of corneal repair rate on day 6 for different mouse groups *P* < 3 × 10^−5^ for MI and MS/MB. *n* = 10 independent mice in Fig. 3d–g. **h** H&E staining images of post-intervention corneas at the alkali burn site. **i** Comparison of epithelial thickness for different mouse groups, *P* < 3.5 × 10^−8^ for MN/MI and MS/MB, *n* = 10 independent samples. All data in (**d**) and (**f**) are presented as means ± SD. In box plots (**e**, **g**, **i**), the dot is the mean, the centerline is the median, box limits are the lower quartile (Q1) and upper quartile (Q3), and whiskers are the most extreme data points that are no more than 1.5 × (Q3 - Q1) from the box limits. Data were analyzed by non-parametric two-sided Mann–Whitney *U* test (**e**) and parametric two-tailed Student’s *t*-test (**g**, **i**). n.s, *, and *** represent nonsignificant (*P* > 0.05), *P* < 0.05 and *P* < 0.001, respectively. Differences were considered significant at *P* < 0.05. MN normal mice without cornea injury, MI mice in the intervention group, MS mice in the sham group, MB mice in the blank control group.

As the anatomical cornea is shown in Fig. 3a^46,47^, the epithelium layer (continuous renewal every 4–7 days) is located on the outer surface of the cornea, followed by a basement membrane above the Bowman’s layer. The intermediate stromal layer contains sparse keratocytes surrounded by connective tissue called collagen fibrils. The Descemet membrane separates the stroma from the underlying endothelium, the innermost corneal layer. The corneal alkali burn (highlighted in red) model is commonly used to study injury-induced pathological changes. Alkali solutions can rapidly penetrate corneal tissues due to their lipophilicity, and the burn grading depends on solution concentrations and injury duration. In this experiment, the moderate corneal alkali burn model (grade II) was selected to study the therapeutic effect of the BPCL, since mild corneal burns (grade I) can be completely self-restituted, and severe burns (grade III and IV) may cause corneal ulcers or perforations (Supplementary Fig. 12a and Supplementary Note 1). In the grade II burn model (Fig. 3b), the corneal epithelial cells were partially missing, and the superficial stromal fibers were irregularly arranged, loose, and structurally disordered. On the other hand, the deep fibers were relatively neat, dense, and regular. Corneal opacity scores (*N* = 0, 1, 2, 3, and 4) in different groups were evaluated over time according to the recognized *Roper-Hall* criteria^48^ (Supplementary Fig. 12b). In our corneal repair experiment, two animal models (mouse and rabbit) were selected to systematically study the repair effect of the BPCL on corneal injury with different relative alkali burn ratios. Considering the low blink rate of mice and rabbits, the injured corneas were intervened with an EF generated by the BPCL every two days under animal anesthesia. The working pressure, frequency, and intervention duration of the BPCL were set at 4 kPa, 1 Hz, and 60 min to simulate human blink motions.

First, the right corneas of mice (C57BL/6) were cauterized with 2-mm diameter alkali (0.15 mol/L NaOH) paper discs for 30 s, and then the eyes were rinsed with 20 ml physiological saline to establish the mouse corneal alkali burn model^49,50^. The corneal injury repair performance was then investigated in four groups fed under the same conditions (Fig. 3c). Mice in the intervention (MI) group were intervened with an EF generated by the BPCL. According to the size of the mouse eye, matching electrodes (Φ ~ 2.2 mm) were designed, and voltage amplitude was adjusted to maintain consistency of horizontal EF strength (~120–420 mV/mm) (Supplementary Fig. 13). Mice in the sham (MS) group were stimulated with deactivated devices in which the electrodes were disconnected from the BPCL. The mice in the blank (MB) control group had no wearable electrodes. Normal mice without cornea injury were labeled as MN_n_ (*n* = 10 independent mice), whose corneas maintained transparency during the 6-day observation period (Fig. 3c). Mice in MI, MS, and MB groups were subjected to the same surgery procedure for corneal injury, and the mice were labeled as MI_n_, MS_n_, and MB_n_ (*n* = 10 independent mice) (Supplementary Fig. 14a). The corneal injury was scored, recorded, and counted once every 2 days after surgery and before euthanasia (Fig. 3d, Supplementary Fig. 14b, Supplementary Fig. 15a, and Supplementary Note 2). On day 0, the corneal opacity (score 3) and epithelial defect appeared rapidly in MI, MS, and MB groups, indicating the moderate corneal alkali burn model was successfully established. For the MI group, the corneal injury area quickly decreased from day 2 to day 4, and the corneal opacity degree was significantly relieved from the opacity of score 3 that partially iris obscured to mild turbidity (score 2). The injured cornea was basically repaired on day 6, marked by the burn trace blurring and almost corneal transparent, along with the opacity score approaching zero. For the MS and MB groups, the corneal opacity area on day 2 was enlarged and more pronounced than what appeared on day 0. Relief of corneal opacity from score 3 to score 2 was observed in the MS and MB groups from day 2 to day 6. However, the corneal defect persisted with moderate turbidity during the entire 6-day monitoring period. In addition, the burn trace did not completely disappear on day 14 without the BPCL intervention (Supplementary Fig. 15b and c). This phenomenon can be attributed to LSCD, in which the limbal epithelial stem cell population is substantially reduced^51^. This chronic condition subsequently leads to corneal conjunctivalization, vascularization, and vision loss. As shown in Fig. 3e, the opacity scores in the MN and MI group on day 6 were 0 and 0.40 ± 0.52, respectively, significantly smaller than those of MS (2.10 ± 0.57) and MB (2.0 ± 0.67) groups. The quantified corneal repair rate over time is shown in Fig. 3f. The repair rates of the MS and MB groups were evidently lower than that of the MI group, and the gap in repair rate between the MI group and control (MS and MB) groups gradually widened from day 2 to day 6. On day 6, the repair rate of the MI group was 96.0 ± 3.4%, significantly higher than those of the control groups (−63.7 ± 88 % for MS and −70.5 ± 67.2% for MB) (Fig. 3g). Corneas at the injury site were collected on day 6 post-intervention for further microscopic analysis. H&E staining analysis confirmed that the MI groups achieved effective re-epithelialization at the injury sites, whereas the MS and MB groups exhibited hypo-epithelialization with a smaller epithelial thickness and cell layer (Fig. 3h and Supplementary Fig. 16). The epithelial thickness on day 6 were 38.10 ± 2.30 μm and 36.84 ± 2.56 μm for MN and MI groups, respectively, which were significantly bigger than those of the control groups (21.90 ± 2.78 μm for MS, and 23.08 ± 3.96 μm for MB) (Fig. 3i). Similar to the MN group, the corneas of the MI group displayed a uniform structure with orderly arranged epithelial cells. Neither histopathological changes nor severe infiltration of inflammatory cells was observed. In contrast, the regenerated epithelium with marked thinning thickness in the untreated control groups (MS and MB) showed an irregular arrangement of epithelial cells. This analysis result confirmed that the pulsed EF from the BPCL could significantly promote orderly corneal injury repair in mice. In addition, three other intervention parameters (intervention frequency, intervention time), including (0.3 Hz, 1h), (0.3 Hz, 2h), and (1 Hz, 2h), were selected to investigate the repair effect in the mouse model (Supplementary Fig. 17 and Supplementary Note 3). The results revealed that there was no statistically significant difference in corneal clarity, repair rate, and epithelial thickness among these intervention groups.

**Fig. 4:**
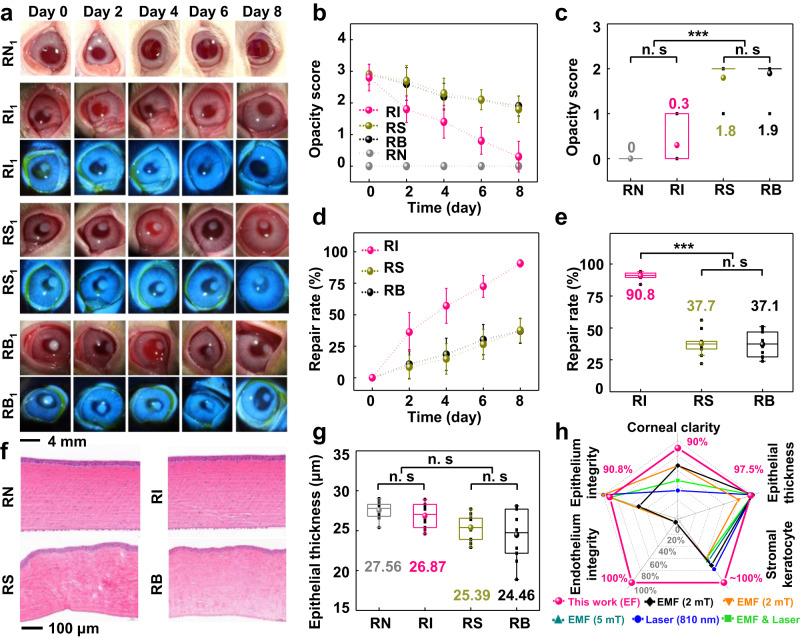
Rabbit corneal alkali burn repair during the BPCL intervention. **a** Serial slit-lamp images of the optical and fluorescent sodium staining (blue) on the alkali burn cornea overtime of the RN, RI, RS, and RB groups (*n* = 10) (Male: *n* = 1–5; Female: *n* = 6–10). **b** Corneal opacity score over time in different rabbit groups. **c** Comparison of corneal opacity on day 8, *P* < 0.00018 for RN/RI and RS/RB. **d** Corneal repair rate over time of the injury area. **e** Comparison of corneal repair rate on day 8 for different rabbit groups, *P* < 2.5 × 10^−12^ for RI and RS/RB. *n* = 10 independent rabbits in Fig. 4b–e. **f** H&E staining images of corneas at the alkali burn site. **g** Comparison of epithelial thickness for different rabbit groups, *n* = 10 independent samples. **h** Comprehensive corneal repair effect of the BPCL compared to the reported results by electromagnetic and laser stimulation (14-day treatment course)^52.53^. Data in (**b**) and (**d**) are presented as means ± SD. In box plots (**c**, **e**, **g**), the dot is the mean, the centerline is the median, box limits are the lower quartile (Q1) and upper quartile (Q3), and whiskers are the most extreme data points that are no more than 1.5 × (Q3 - Q1) from the box limits. Data were analyzed by non-parametric two-sided Mann–Whitney *U* test (**c**) and parametric two-tailed Student’s *t*-test (**e**, **g**). n.s and *** represent nonsignificant (*P* > 0.05) and *P* < 0.001, respectively. Differences were considered significant at *P* < 0.05. RN normal rabbits without cornea injury, RI rabbits in the intervention group, RS rabbits in the sham group, RB rabbits in the blank control group, EF electric field, EMF electromagnetic filed, mT millitesla.

After confirming the positive therapeutic effect in mice, the injury repair in rabbits was further investigated to simulate the actual size of the human eye and circumvent the LSCD effect. Identical to the grouping of mice, the rabbits were divided into four groups (RN, RI, RS, and RB) and labeled as RN_n_, RI_n_, RS_n_, and RB_n_ (*n* = 10 independent rabbits). Corneas in the RN group were totally clear during the 8-day observation period (Fig. 4a and Supplementary Fig. 18). Rabbits in RI, RS, and RB groups underwent the same surgical procedure as the mice, and the corneal repair was monitored and scored every 2 days (Fig. 4a, Supplementary Fig. 14b, and Supplementary Fig. 18). The injury boundaries were clear throughout the observation process, and the corneal injury area did not expand in RI, RS, and RB groups, indicating that LSCD was avoided in the rabbit model. Serial slit-lamp images of the optical and fluorescent sodium staining (blue) demonstrated that the corneal epithelial defect healed rapidly, and corneal opacity was significantly relieved under the BPCL intervention. In contrast, rabbits in RS and RB groups showed delayed corneal repair (Fig. 4b). Opacity scores on day 8 were 0, 0.30 ± 0.48, 1.80 ± 0.42, and 1.90 ± 0.31 for RN, RI, RS, and RB groups (Fig. 4c). The quantified corneal repair rate over time is shown in Fig. 4d. The gap in repair rate between the RI group and control groups gradually widened from day 2, and the repair rates of the RS and RB groups were remarkably lower than that of the RI group. On day 8, the repair rate of the RI group was 90.8 ± 2.9%, significantly higher than those of the control groups (37.7 ± 9.7% for RS and 37.1 ± 9.8% for RB) (Fig. 4e). Furthermore, the burn trace did not completely disappear on day 18 without the BPCL intervention (Supplementary Fig. 19). On day 8 post-intervention, corneal tissue at the injury sites was collected for histological examination by H&E staining (Fig. 4f and Supplementary Fig. 20). The mean epithelial thickness on day 8 in the RN, RI, RS, and RB groups were 27.56 ± 1.09 μm, 26.87 ± 1.54 μm, 25.39 ± 1.60 μm, and 24.46 ± 3.16 μm, respectively. Unlike the mouse model, there was no significant difference in epithelial thickness and cell layer among the four groups (Fig. 4g and Supplementary Fig. 20). Histological analysis revealed that corneas treated by the BPCL were similar to those in the RN group. The corneas in RI group re-epithelialized uniformly and orderly, presenting intact neo-epithelium tightly connected to the underlying stromal tissue. In contrast, control groups (RS and RB) had irregular regenerating epithelium with uneven thickness, significant stromal keratocyte loss, and undesirable architecture distortion. In addition, compared to the reported nonpharmacological electromagnetic and laser therapeutic approaches based on similar rabbit models (14-day treatment course)^52,53^ (Fig. 4h), our correlated BPCL system demonstrated outstanding comprehensive repair effect under the evaluation criteria of the corneal clarity recovery, epithelial integrity and thickness, stromal keratocyte loss, and endothelium integrity. Remarkably, the corneal clarity recovery (90%) intervened by the BPCL with a much shorter treatment time vastly surpassed the performance of other reported physical methods. These animal results revealed that the enhanced endogenous EF by the BPCL could accelerate orderly corneal repair in mice and rabbits (Supplementary Fig. 21).

### BPCL intervention mechanisms for effective corneal repair

**Fig. 5:**
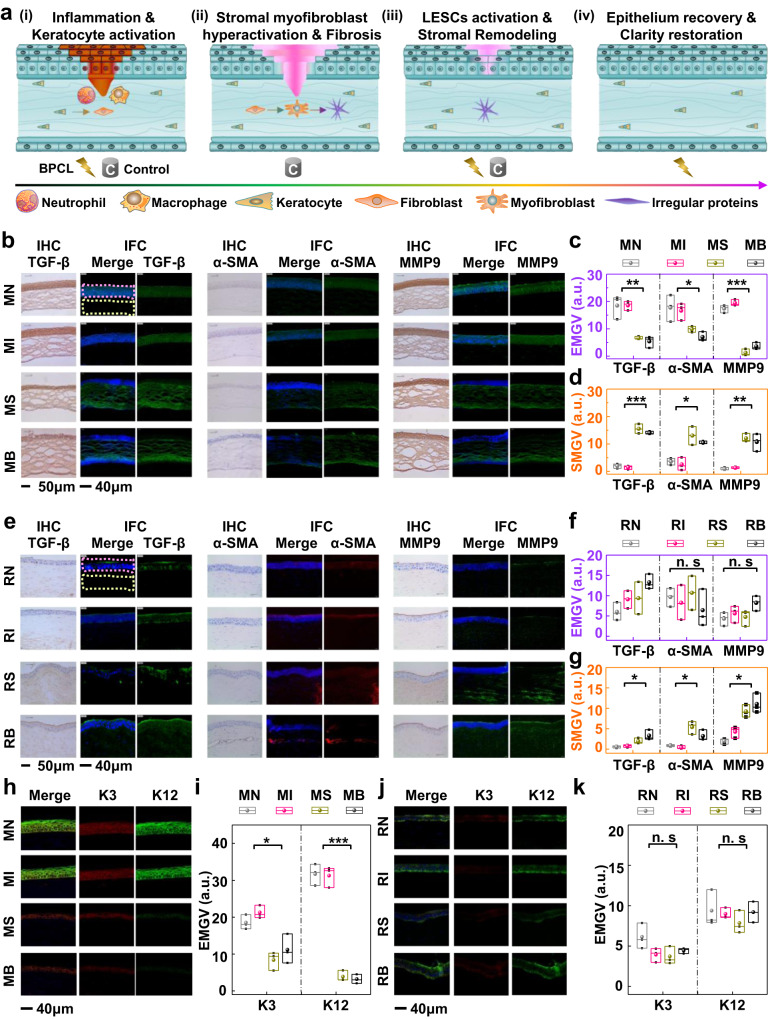
Biological intervention mechanism of the BPCL. **a** Stages in corneal alkali burn repair include inflammation and keratocyte activation (i), stromal myofibroblast hyperactivation and fibrosis (ii), LESCs activation and stromal remodeling (iii), corneal repair and clarity restoration (iv). **b** Mouse IHC and IFC staining images of multiple growth factors, including TGF-*β*, *α*-SMA, and MMP9. **c** EMGV of mice, P < 0.01 for TGF-*β*, P < 0.05 for *α*-SMA, P < 2.4 × 10^−4^ for MMP9, *n* = 3 independent samples. **d** SMGV of mice, *P* < 2.6 × 10^−4^ for TGF-*β*, *P* < 0.012 for *α*-SMA, *P* < 0.008 for MMP9, *n* = 3 independent samples. **e** Rabbit IHC and IFC staining images of multiple growth factors, including TGF-*β*, *α*-SMA, and MMP9. **f** EMGV and **g** SMGV of rabbits, *P* < 0.05 for TGF-*β*, *P* < 0.045 for *α*-SMA, *P* < 0.015 for MMP9, *n* = 3 independent samples. **h** Mouse IFC staining images of growth factors including K3 and K12. (i) EMGV of mice K3 and K12, *P* < 0.047 for K3, *P* < 1.3 × 10^−4^ for K12, *n* = 3 independent samples. **j** Rabbit IFC staining images of growth factors including K3 and K12. **k** EMGV of rabbit K3 and K12, *n* = 3 independent samples. In box plots (**c**, **d**, **f**, **g**, **i**, **k**), the dot is the mean, the centerline is the median, box limits are the lower quartile (Q1) and upper quartile (Q3), and whiskers are the most extreme data points that are no more than 1.5 × (Q3 - Q1) from the box limits. Data were analyzed by parametric two-tailed Student’s *t*-test, n.s, *, **, and *** represent nonsignificant (*P* > 0.05), *P* < 0.05, *P* < 0.01, and *P* < 0.001, respectively. Differences were considered significant at *P* < 0.05. LESCs limbal epithelial stem cells, MN normal mice without cornea injury mice in the intervention group, MS mice in the sham group, MB mice in the blank control group, RN normal rabbits without cornea injury, RI rabbits in the intervention group, RS rabbits in the sham group, RB rabbits in the blank control group, EMGV mean gray value of epithelium, SMGV mean gray value of stroma, a. u. arbitrary units, TGF-*β* transforming growth factor beta, *α*-SMA alpha-smooth muscle actin, MMP9 matrix metalloproteinase 9, IHC immunohistochemistry, IFC immunofluorescence, K3 Keratin 3, K12 Keratin 12.

As the schematic diagram is shown in Fig. 5a, the corneal repair response to alkali burn occurs in four overlapping but distinct cascade stages^54^: (i) inflammation and keratocyte activation, (ii) stromal myofibroblast hyperactivation and fibrosis, (iii) limbal epithelial stem cells (LESCs) activation and stromal remodeling, (iv) epithelium recovery and clarity restoration. During the inflammation stage (repair stage i), neutrophils and macrophages are recruited, which produce inflammatory cytokines and chemokines to induce the transdifferentiation of normally quiescent keratocytes into fibroblasts, thereby facilitating corneal repair. In the stage of stromal myofibroblasts hyperactivation and fibrosis (repair stage ii), corneal injury involving disruption to the Bowman membrane results in the increased exposure of cytokines and growth factors, including transforming growth factor beta (TGF-*β*)^55,56^, in the anterior stroma. Fibroblasts further transdifferentiated into myofibroblasts, marked by stromal alpha-smooth muscle actin (*α*-SMA)^56,57^. In pathological conditions without external intervention, stromal hyperactivated myofibroblasts persist and secrete excessive amounts of irregular extracellular matrix proteins, resulting in corneal fibrosis and opacity. The LESCs activation and stromal remodeling stage (repair stage iii) include limbal cellular proliferation and migration, epithelium injury repair to restore the barrier function, and myofibroblasts disappearing due to apoptosis. The stromal inflammation degree and disorganized stromal remodeling were positively correlated with the expression of matrix metalloproteinases (MMPs)^54,58^. Finally, the epithelium recovery is complete, and tissue transparency is maintained under effective intervention (repair stage iv), whereas tissue fibrosis may progress with long-term corneal opacity in untreated corneas.

To understand the mechanism of accelerated corneal injury repair intervened by the BPCL, multiple growth factors involved in corneal repair, including TGF-*β*, *α*-SMA, and MMP9, were investigated and evaluated. To assess the distribution of these key growth factors, corneas at injury sites from different animal groups were collected and analyzed by immunohistochemistry (IHC) and immunofluorescence (IFC) at the end of the BPCL intervention (Fig. 5b, e, and Supplementary Fig. 22a and b). IHC staining images qualitatively showed an overall expression distribution of various growth factors in two models, where the dark brown color represented the expression of corresponding growth factors. IFC staining was employed to further quantify TGF-*β*, *α*-SMA, and MMP9 expression levels based on their fluorescent intensities. The mean gray value (IntDen/Area) of epithelium and stroma from ImageJ V 1.8.0 software statistics were defined as EMGV (purple border) and SMGV (yellow border), respectively (Fig. 5c, d, f, g).

In the mouse model, the IHC results showed the epithelial secretion of TGF-*β*, *α*-SMA, and MMP9 in MI and MN groups were significantly enhanced compared to the control groups (MS and MB), while the trends in secretion intensity were reversed in the stroma (Fig. 5b). A similar phenomenon was found in IFC results. For the MI and MN groups, only TGF-*β* (green), *α*-SMA (green), and MMP9 (green) in the corneal epithelium were stained positively, and expression of growth factors was almost absent in the stroma^55,56^. The average EMGV of TGF-*β*, *α*-SMA, and MMP9 were (18.51, 17.73, and 17.40) and (18.57, 16.62, and 19.43) for MN and MI groups, respectively, statistically significantly higher than those of MS (6.62, 9.90, and 1.23) and MB (5.40, 6.98, and 3.58) groups (Fig. 5c). In corneal epithelium, TGF-*β*, *α*-SMA, and MMP9 were required for inherent epithelial renewal and physiological injury repair, since they were indispensable growth factors for normal metabolism and migration of the corneal epithelial cells. For the untreated mice, the weak expression of growth factors in the epithelium resulted in a small epithelial thickness and irregular epithelial arrangement (Fig. 3h, i). In contrast, strong TGF-*β*, *α*-SMA, and MMP9 expressions were detected in the corneal stroma of the MS and MB groups. The average SMGV of TGF-*β*, *α*-SMA, and MMP9 were (2.01, 3.61, and 0.88) and (1.47, 2.54, and 1.32) for MN and MI groups, respectively, statistically significantly lower than those of MS (15.52, 13.04, and 12.05) and MB (14.0, 10.46, and 10.73) groups (Fig. 5d). In the stroma, TGF-*β* and *a*-SMA were the essential inducer and primary phenotype of myofibroblast transformation, respectively. The MMP9 represented stromal inflammation and disorganized remodeling. Enhanced expression of these growth factors led to the fibrotic stroma, and thus the cornea remained long-term turbidity.

For the rabbit model, the epithelial IHC results showed that there was no noticeable difference in intensity of TGF-*β*, *α*-SMA, and MMP9 in four different groups, while the stromal secretion of growth factors in group RS and group RB were significantly enhanced compared to the RI and RN groups (Fig. 5e). Similarly, the average EMGV of TGF-*β* (green), *α*-SMA (red), and MMP9 (green) were (6.01, 9.66, and 4.32), (9.11, 8.26, and 5.62), (9.38, 10.7, and 4.77), (13.28, 6.41, and 8.21) for RN, RI, RS and RB groups, respectively. EMGV values were comparable across four different groups (Fig. 5f). Therefore, the thickness of the epithelium was not significantly different for various groups. However, the capacity of endogenous EF alone to arrange the epithelial cells in an orderly manner was insufficient, resulting in uneven epithelial thickness in RS and RB groups (Fig. 4f, g). The average SMGV of TGF-*β*, *α*-SMA, and MMP9 were (0.59, 0.87, and 2.01) and (0.79, 0.41, and 4.28) for RN and RI groups, respectively, statistically significantly lower than those of RS (1.95, 5.41, and 9.26) and RB (3.32, 3.2, and 11.06) groups (Fig. 5g). Due to the strong expression of growth factors in the stroma, myofibroblasts transdifferentiated from keratocytes rapidly produced excessive amounts of irregular proteins. In addition, tractional forces were exerted across these irregular proteins, resulting in the stromal architecture distortion and corneal opacity (Fig. 4f).

Two growth factors, including Keratins 3 and 12 (K3 and K12), were further investigated to understand the epithelial differentiation and maturation mechanism intervened by the BPCL. In particular, it was noted that both K3 and K12, specific markers for corneal epithelial differentiation and maturation^59–61^, were not expressed in the stroma. As IFC staining images shown in Fig. 5h, j (Supplementary Fig. 22c), only K3 (red) and K12 (green) in the corneal epithelium were stained positively, and expression of growth factors was almost absent in the stroma. In the mouse model, the IFC results showed the epithelial secretion of K3 and K12 in MI and MN groups were significantly enhanced compared to the control groups (MS and MB) (Fig. 5h). The average EMGV of K3 and K12 were (18.52 and 31.69) and (21.28 and 31.28) for MN and MI groups, respectively, statistically significantly higher than those of MS (8.38, and 3.87) and MB (11.18 and 3.20) groups (Fig. 5i). For the mouse model, the BPCL intervention could promote differentiation and maturation of the epithelium, leading to effective re-epithelialization at the injury sites. For the rabbit model, the epithelial IFC results showed that there was no noticeable difference in intensity of K3 and K12 in four different groups (Fig. 5j). The average EMGV of K3 and K12 were (6.13 and 9.38), (3.93 and 8.98), (3.72 and 7.85), (4.45 and 9.20) for RN, RI, RS and RB groups, respectively. Though EMGV values were comparable across four different groups (Fig. 5k), rabbit control groups (RS and RB) had irregular regenerating epithelium with uneven thickness and undesirable architecture distortion. In general, the BPCL intervention could promote the corneal epithelium arrangement, differentiation, and maturation.

## Discussion

Growth factors (TGF-*β*, *α*-SMA, and MMP9) play a dual role in corneal injury repair^55^. On the one hand, stromal myofibroblast activity induced by these growth factors persists, leading to chronic tissue contracture, architecture distortion, and corneal opacity. On the other hand, they are indispensable for epithelial inherent renewal and metabolism. In our experiment, the BPCL intervention could act as a critical regulatory factor that limits the fibrotic response by modulating the entry of epithelial-derived growth factors into the stroma, which could rapidly activate LESCs while suppressing myofibroblast hyperactivation and stromal fibrosis. Therefore, corneal repair under the BPCL intervention primarily underwent repair stages i, iii, and iv, while the untreated control group mainly underwent stages i, ii, and iii (Fig. 5a). For control groups in mouse (MS and MB) and rabbit (RS and RB) models, corneal epithelial thickness and the relative distribution of growth factors were slightly different. This phenomenon can be attributed to a larger relative corneal burn ratio in mice induced a relatively serious LSCD and delayed the repair progress. Corneal injury repair requires wound closure, epithelial thickness restoration, and orderly tissue (stroma and epithelium) arrangement and differentiation, and there is no necessary connection between repair rate and epithelial thickness improvement. In addition, both models were tested for a vascular endothelial growth factor (VEGF) by IHC staining and no positive expression in the entire cornea, indicating no vascularization in the corneal injury site (Supplementary Fig. 23).

Here, we proposed a snowflake-inspired BPCL consisting of a fractal piezoelectric electret film and a serpentine EF generation circuit. The structural design and performance optimization could render desirable mechanical compliance and convert tiny daily blink motions (pressure ~ 4 kPa) into an adequate unidirectional EF (>120 mV/mm) to accelerate corneal alkali burn injury repair. The therapeutic verification was achieved on the mouse and rabbit models. Considering the dual role of growth factors (TGF-*β*, *α*-SMA, and MMP9), the BPCL intervention was advantageous in blocking downstream signaling pathways specifically involved in myofibroblast transdifferentiation whilst preserving and harnessing the signaling pathways required for homeostasis and normal metabolism. As an intervention result, the mice’ corneal clarity, repair rate, and epithelial thickness improved by more than 53%, 159%, and 36%, respectively. The rabbits’ corneal clarity and repair rate increased by over 50% and 52%, respectively. This outstanding corneal therapeutic effect outperformed other reported nonpharmacological approaches based on similar corneal alkali burn models. In general, the BPCL can alleviate the inflammatory fibrosis, lessen the corneal opacification, promote the orderly repair effect, and improve patient compliance. More broadly, this work proposed design criteria for effective corneal therapeutic approaches by correlating endogenous microenvironment modulation to orderly injury repair.

## Methods

### Ethics approval

All experiments performed with animals and human participants were conducted under a standard protocol (1061420210617007) approved by the Ethics Committee of the Animal Experiment Center of University of Electronic Science and Technology of China.

### Fabrication of the BPCL

Fabrication procedures of the BPCL were displayed in Supplementary Fig. 1. First, the piezoelectric polypropylene electret film (~84 μm, Shanghai Electret Materials Technology Co., Ltd) with double-sided aluminum (Al) electrodes (~100 nm) and ITO film (~100 nm) sputtered on the PET substrate (~23 μm, Hefei Kejing Material Technology Co., Ltd) were prepared. Then, Al/electret/Al and ITO/PET films were adhered to the water-soluble tape (Shenzhen Yueda New Material Co., Ltd). The Al/electret/Al film and ITO/PET film were patterned to dendritic fractal star polygon and plate-like serpentine structures by femtosecond laser cutting technology (laser power~2.0 W, Design Comes Ture Technology Co., Ltd, DirectLaser PC6), respectively (Supplementary Fig. 1a). Next, as shown in Supplementary Fig. 1b (left), the polyimide (PI) film (~25 μm, Suzhou Yingchuan New Material Technology Co., Ltd) on a glass slide was also patterned by laser cutting to fabricate a template. Then PDMS solution (Dow Corning 184, 10:1, Dongguan Sanbang New Material Technology Co., Ltd) was spin-coated on the template, acting as the bottom hydrophobic encapsulation layer of the PEG (Supplementary Fig. 1b, right). Next, the piezoelectric film and concentric circular ITO electrodes were successively adhered to the template-based PDMS substrate by transfer printing technique according to bilayer alignment marks, followed by the water-soluble tape removed (Supplementary Fig. 1c). Finally, the ITO electrodes and the PEG component were connected via the micro rectifier (RisymB5819WT Schott diodes, ~2 × 1.6 × 0.4 mm^3^, Shenzhen Xuanxinwei Technology Co., Ltd) with PDMS encapsulation. Then the peeled device was packaged by hydrophilic pHEMA (Shanghai Merck Life Sciences Technology Co., Ltd, Sigma-Aldrich) in a mold with 365 nm light irradiation for ~10 minutes^36^ (Supplementary Fig. 1d).

### Oxygen permeability measurement

A gas permeability test system (GTR 701-M, Chengde Tuowei Technology Co., Ltd) was employed to measure the oxygen permeability coefficient based on the differential pressure method^62,63^ (Supplementary Fig. 3a). Oxygen permeability coefficients of PDMS and pHEMA films (area: 10 × 10 cm^2^, thickness~50μm) were measured in the differential pressure range from 0~1200 Pa. The test ambient temperature and humidity are ~34 °C and ~24.6 %RH, respectively. As shown in Supplementary Figure 3b, oxygen passing through the film material is a penetration process. From a microscopic perspective, there are two main steps: (i) the oxygen is dissolved, adsorbed on one side of the film surface, and reaches dissolution balance on the surface layer. Then the oxygen diffuses to the other side due to the oxygen concentration gradient on both sides of the film. (ii) The gas reaching the surface on the other side of the film is desorbed or released until the concentration gradient on both sides reaches an equilibrium state.

### FEA mechanical characterization

The FEA mechanical properties of the BPCL and the PEG with different *D*_*n*_ (4, 6, 8, 12, and 30) were evaluated by the ABAQUS 6.12 software. According to the values inherent in the software, the values of (Young’s modulus, Poisson’s ratio) were set to (69 GPa, 0.35), (4.5 GPa, 0.30), (118 GPa, 0.35), (2 MPa, 0.49) for Al electrode, polyethylene terephthalate film, ITO electrode, and PDMS, respectively. The scattering of Young’s modulus values reflects different densities and structures of piezoelectric polypropylene films. It correlates with their electromechanical properties: the lower Young’s modulus, the higher the piezoelectric coefficient. Young’s modulus and Poisson’s ratio of the piezoelectric polypropylene film are ~1.5 MPa^64,65^ and 0.34^66^ as the piezoelectric coefficient is ~145 pC/N. Young’s modulus and Poisson’s ratio are ~5 MPa^67^ and 0.48^68^ for the pHEMA. The fractal structure and serpentine lines were refined to ensure computational accuracy. The strain distribution of the BPCL (*D*_*n* _= 12) was analyzed by a pressing system under a series of pressing heights (from 0 to 10 mm) using a spherical ball with a radius of 6 mm. The strain (≤1.0%) subjected by the BPCL was smaller than the failure strain (5%) even when the BPCL was pressed up to 10 mm (Fig. 1g and Supplementary Fig. 4). Similarly, the strain (≤1.0%) of PEG (*D*_*n* _= 12) was smaller than the failure strain (5%) when the PEG was biaxially stretched to 40% (Supplementary Fig. 7c).

### In vitro and in vivo biocompatibility validation of the BPCL

Mouse fibroblast 3T3 cells (SCSP-5038) were obtained from the Cell Bank, Shanghai Institutes for Biological Sciences, Chinese Academy of Sciences. Mouse fibroblast 3T3 cells were first cultured in vitro on the PDMS/pHEMA hybrid encapsulation layer and a reference dish for three days. They both underwent the same procedure and were raised under the same conditions. Cells were cultured at 37 °C in a humidified incubator with 5% CO_2_ in an α-MEM medium with 10% fetal bovine serum (Gemini, Australia origin, Shanghai Jinpin Chemical Technology Co., Ltd) and 1% penicillin/streptomycin (P1400, Beijing Solarbio Science & Technology Co., Ltd). After incubation for 1, 2, and 3 days, the sample was first fixed with 2 to 4% formaldehyde for 15 min and rinsed three times with prewarmed phosphate-buffered saline (PBS, Shanghai Merck Life Sciences Technology Co., Ltd, Sigma-Aldrich). Then, fibroblast 3T3 cells were imaged using a confocal microscope after staining with Texas Red-X Phalloidin (100 nM, Thermo Fisher Scientific) and Hoechst (50 nM, Shanghai Merck Life Sciences Technology Co., Ltd, Sigma-Aldrich). (Fig. 1h). In addition, to assess the relative viability of 3T3 cells, the working culture medium was also transferred to a 96-well plate, and 100 μL of 3-{4,5-dimethylthiazol-2-thiazolyl}-2,5-diphenyl-2H-tetrazolium bromide solution (Shanghai Aladdin Biochemical Technology Co., Ltd) was added to each well. The medium was removed after 4-hour incubation, and dimethyl sulfoxide (500 μL/well, Shanghai Merck Life Sciences Technology Co., Ltd, Sigma-Aldrich) was added to dissolve the precipitated fomazan. The optical density of the solution was evaluated at a wavelength of 490 nm using a microplate spectrophotometer (Fig. 1i). In vivo biocompatibility validation was performed on a rabbit. The optical images indicated that the BPCL was associated without corneal tissue injury throughout the 2-week observation process (Supplementary Fig. 5a). In addition, the H&E staining image showed that neither histopathological changes nor severe infiltration of inflammatory cells was observed (Supplementary Fig. 5b).

### Electrical characterization and EF simulation of the BPCL

The electrical signals of all devices was tested by a Keithley 6514 electrometer (USA, internal impedance is 200 TΩ) and a portable oscilloscope (Agilent, DSO-X2012A). The voltage signals shown in Fig. 2c (25 kPa), d, f, and h, Supplementary Figs. 8, 10, and 11, and Supplementary Movie 4 were measured by a computer-controlled linear motor (LinMot-B01-37X166/160, Nanjing Huiyanda Electric Co., Ltd). The pressure was calibrated with a commercial pressure transducer. The voltage signals in Fig. 2g, Supplementary Fig. 9, and Supplementary Movie 3 were monitored in vivo during blink in the human-worn state. In the simulation domain, the Ansys Maxwell finite element solver (AMFES) and the electrostatic solution type were employed to assess EF distributions simulation. In particular, the salt water model with permittivity 81 was used to simulate tissues and body fluids (Fig. 2i, j, and Supplementary Fig. 13d).

### Human research participants

All participation in the study is entirely voluntary. The voltage output in the human-worn state was monitored for proof-of-principle testing, and the non-invasive test did not involve tissue damage or biological characterization. All volunteers were recruited within the University of Electronic Science and Technology of China, for a duration of 30 min with a compensation of $50. This recruitment excluded individuals with low tolerance to wearing contact lenses. Three healthy subjects participated in voltage monitoring, including two men (Participant I: 23 years old; Participant II: 26 years old) and one woman (24 years old). Subjects have read the relevant research materials and received satisfactory answers to all questions. Subjects fully understand the relevant medical research materials and the potential risks and benefits of the research. The subjects know that participating in the study is voluntary and they have the right to withdraw at any time. The subjects agree to the review of research materials by the drug regulatory department, ethics committee, or applicant and have expressed their willingness to participate in the study. The subjects agree to publish research results (including age, sex, and Supplementary Movie 3) in scientific journals or presentations at scientific conferences. All experiments performed with human participants were conducted under a standard protocol (1061420210617007) approved by the Ethics Committee of the Animal Experiment Center of University of Electronic Science and Technology of China.

### Mice and rabbits

Eight-week-old C57BL/6 mice and eight-week-old New Zealand white rabbits (*n* = 10; Male: *n* = 1–5, Female: *n* = 6–10; Chengdu Dossy Experimental Animals Co., Ltd; Both male and female animals were considered and employed to increase statistical robustness.) were subjected to the same corneal injury procedure and fed under the same conditions (Supplementary Fig. 14a). All animals were housed in individual cages in a temperature-controlled (22 °C) room (relative humidity: 45% ~ 60%) with a 12-h light/12-h dark cycle and provided free water and feed. All animal-based procedures were performed under the National Institutes of Health guidelines for the care and use of laboratory animals. All animal experiments were conducted under a protocol (1061420210617007) approved by the Ethics Committee of the Animal Experiment Center of University of Electronic Science and Technology of China.

### Corneal modeling and BPCL experimental setup

The anesthesia was first induced by inhalation of 2–5% isoflurane and then maintained with 2% isoflurane (Shenzhen RWD Life Technology Co., Ltd). Mice and rabbits were immobilized in the prone position after anesthesia. The right corneas were cauterized with 2-mm diameter alkali (0.15 mol/L NaOH, Shanghai Merck Life Sciences Technology Co., Ltd, Sigma-Aldrich) paper discs for 30 s, and then the eyes were rinsed with 20 ml physiological saline (0.9 g/mL NaCl, Shanghai Merck Life Sciences Technology Co., Ltd, Sigma-Aldrich) to establish the corneal alkali burn model. Under animal anesthesia, the injured corneas were intervened with an EF generated by the BPCL (1 Hz, 60 min) once every 2 days. The corneal injury was scored after surgery and before euthanasia according to the *Roper-Hall* criteria. For the rabbit model, the intervention voltage was ~0.7 V induced by a pressure of 4 kPa. For the mouse model, matching electrodes (Φ ~ 2.2 mm) were designed, and voltage amplitude was adjusted to maintain the consistency of horizontal EF strength (Supplementary Fig. 13). All animal experiments were performed following the standard protocol (1061420210617007) approved by the Ethics Committee of the Animal Experiment Center of University of Electronic Science and Technology of China.

Corneal repair calculation methods. During corneal injury repair process, the relationship between the actual injury area (*S*_*I*_) area and the observed injury area (*S*_*O*_) on cross-sectional mouse and rabbit eyeballs was shown in Supplementary Fig. 14b and Supplementary Note 2. The corneal repair rate, clarity recovery, epithelial thickness restoration, and cell layer restoration were defined as Eqs. (1), (2), (3), and (4)^69^:

1 $${Corneal}\,{repair}\,{rate}\, \left(\%\right)={(S_{{I \, Day \, }0}{-S}_{{I \, Day \, x}})/S}_{{I \, Day \, }0} \times \,100$$

2 $${Clarity}\,{recovery}\, \left(\%\right)={(C_{{Day \, }0}{-C}_{{Day \, x}})/C}_{{Day \, }0} \times 100$$

3 $${Thickness}\,{restoration}\, \left(\%\right)=1-({T}_{N{ormal}}{-T}_{{Day \, x}}){/T}_{{Normal}} \times 100$$

4 $${Cell}\,{layer}\, {restoration}\, \left(\%\right)=1-({CL}_{{Normal}}{-{CL}}_{{Day \, x}})/{CL}_{{Normal}} \times 100$$

where *S*_*I*_, *C*, *T*, and *CL* are the actual corneal injury area, corneal opacity score, epithelial thickness, and the epithelial cell layer, respectively, and *x* is the day after the corneal alkali burn.

### H&E staining of mouse and rabbit corneas

Tissues at the corneal injury sites in both animal models were collected post-intervention for histological examination by H&E staining. Normal corneas without injury in MN and RN groups were also collected at the same time points. Tissues were fixed with 4% formaldehyde (Shanghai Merck Life Sciences Technology Co., Ltd, Sigma-Aldrich), and sections were prepared at 3 μm for H&E staining. H&E slides were observed and photographed using an inverted optical microscope (Figs. 3b, h, 4f, Supplementary Fig. 16a, Supplementary Figs. 17a, 19c, and 20a).

### Immunohistochemistry and Immunofluorescence staining of corneas

The collected corneas were fixed in 10% formaldehyde solution (Shanghai Merck Life Sciences Technology Co., Ltd, Sigma-Aldrich) for 48 h and decalcified with 5% nitric acid (Shanghai Merck Life Sciences Technology Co., Ltd, Sigma-Aldrich). After dehydration and washing, corneas were embedded in paraffin. For each specimen, cut consecutive sections parallel to the injured corneal region using a microtome. Immunohistochemical (IHC) staining was performed to evaluate the level of TGF-*β*, *α*-SMA, and MMP9 with corresponding antibodies. For immunofluorescence (IFC) staining, 10 μm thick frozen corneal samples were fixed with cold acetone (Shanghai Merck Life Sciences Technology Co., Ltd, Sigma-Aldrich) for 5 min, washed with cold PBS, and sealed with 2% donkey serum (Shanghai Merck Life Sciences Technology Co., Ltd, Sigma-Aldrich) at room temperature for 1 h. Then, slices were incubated with rabbit/mouse TGF-*β* antibody, *α*-SMA antibody, MMP9 antibody, Keratin 3 antibody, and Keratin 12 antibody overnight at 4 °C. After washing three times with cold PBS, slices were then stained with Alaxa488-labeled Donkey-anti-rabbit/mouse antibody for 1 h. After three rounds of washing with cold PBS, all slides were mounted with a mounting medium with DAPI (Shanghai Santa Cruz Biotechnology Co., Ltd) and covered with cover slides for imaging using a confocal microscope (Fig. 5b, e, h, j and Supplementary Fig. 22). Antibodies: Anti-TGF-*β*-APC (Biolegend, Clone TW7-16B4, Catalog: 141406, Dilution: 1:400, Mouse Antibody), Anti-*α*-SMA-antibody (Biolegend, Clone 1A4, Catalog: 614852, Dilution:1:500, Mouse Antibody), Anti-TGF-*β*-antibody (Abcam, ab215715, Dilution: 1:500, Rabbit Antibody), Anti-*α*-SMA-antibody (Abcam, ab5694, Dilution: 1:200, Rabbit Antibody), Anti-MMP9-antibody (Abcam, ab283575, Dilution: 1:500, Mouse/Rabbit Antibody), Anti-Keratin 3-antibody (Abcam, ab77869, Dilution: 1:200, Mouse/Rabbit Antibody), Anti-Keratin 12-antibody (Abcam, ab185627, Dilution: 1:1000, Mouse/Rabbit Antibody).

### Statistics and reproducibility

OriginPro 9 was used for data analysis, plotting and statistics. Corneal clarity data were analyzed by non-parametric two-sided Mann–Whitney *U* test^70,71^. Two-tailed unpaired Student’s *t*-tests performed statistical analysis for repair rate, epithelial thickness, corneal epithelial cell layer, and IFC intensity^70^. In box plots, the dot is the mean, and the centerline is the median, the box limits are the lower quartile (Q1) and upper quartile (Q3), and whiskers are the most extreme data points that are no more than 1.5 × (Q3 - Q1) from the box limits. n.s, *, **, and *** represent nonsignificant (*P* > 0.05), *P* < 0.05, *P* < 0.01, and *P* < 0.001, respectively. In general, *P* value < 0.05 indicates a significant difference. For representative experiments (Figs. 1c, h; 3b, h; 4f; Supplementary Figs. 2d; 5b; 11c, d; 19c; 22a, b, c; 23a, b), each experiment was repeated independently many times (≥3) with similar results, demonstrating good data reproducibility.

### Reporting summary

Further information on research design is available in the Nature Portfolio Reporting Summary linked to this article.

## Supplementary information

Supplementary Information

Description of additional supplementary files

Supplementary Movie 1

Supplementary Movie 2

Supplementary Movie 3

Supplementary Movie 4

Supplementary Movie 5

Reporting Summary

## Data Availability

The authors declare that all data supporting the findings of this study are available within the Article and its Supplementary Information. Any additional requests for information can be directed to, and will be fulfilled by, the corresponding authors. Source data are provided with this paper.

## Source data

Source data
