# Supplementary material for: Snowflake-inspired and blink-driven flexible piezoelectric contact lenses for effective corneal injury repair
Source: Nat Commun. 2023 Jun 17;14:3604. doi: 10.1038/s41467-023-39315-6 (PMC10276863; doi:10.1038/s41467-023-39315-6)
Supplement: Supplementary file 1 — Supplementary Information [file 41467_2023_39315_MOESM1_ESM.pdf]

Supplementary Information for

Snowflake-inspired and blink-driven flexible piezoelectric contact lenses for effective corneal injury repair

Guang Yao[1,2,3]*, Xiaoyi Mo[1], Shanshan Liu[4], Qian Wang[1], Maowen Xie[1], Wenhao Lou[1], Shiyan Chen[5], Taisong Pan[1], Ke Chen[4,5]*, Dezhong Yao[4], Yuan Lin[1,2,6]*

[1]School of Materials and Energy, University of Electronic Science and Technology of China, Chengdu 610054, Sichuan, China.

[2]State Key Laboratory of Electronic Thin films and Integrated Devices, University of Electronic Science and Technology of China, Chengdu 610054, Sichuan, China.

[3]Shenzhen Institute for Advanced Study, University of Electronic Science and Technology of China, Shenzhen 518110, China

[4]MOE Key Laboratory for Neuroinformation, The Clinical Hospital of Chengdu Brain Sciences Institute, University of Electronic Science and Technology of China, Chengdu 610054, Sichuan, China.

[5]Department of Ophthalmology, Sichuan Academy of Medical Sciences and Sichuan Provincial People's Hospital, Medical School, University of Electronic Science and Technology of China, Chengdu 610054, Sichuan, China.

[6]Medico-Engineering Cooperation on Applied Medicine Research Center, University of Electronic Science and Technology of China, Chengdu 610054, Sichuan, China.

*Correspondence should be addressed to G.Y. (gyao@uestc.edu.cn), K.C. (chenke0703@uestc.edu.cn) or Y.L. (linyuan@uestc.edu.cn)

Table of Contents

Supplementary Notes 3

Supplementary Note 1 | The cellular layers of the corneal epithelium........ 3

Supplementary Note 2 | The corneal repair rate calculation........ 3

Supplementary Note 3 | Mouse cornea repair with different intervention parameters........ 4

Supplementary Figures 6

Supplementary Figure 1 | The fabrication procedure of the BPCL 6

Supplementary Figure 2 | Characterization of the BPCL 8

Supplementary Figure 3 | Oxygen permeability characterization 9

Supplementary Figure 4 | Mechanical robustness of the BPCL 10

Supplementary Figure 5 | *In vivo* biocompatibility verification of the BPCL........ 11

Supplementary Figure 6 | Fractal star polygon structure of the BPCL........ 12

Supplementary Figure 7 | Performance of the PEGs with a different number of dendrites. . 13

Supplementary Figure 8 | Radar chart of different PEGs for performance comparison 15

Supplementary Figure 9 | Performance comparison of the BPCLs (D_n =12 and 30). 16

Supplementary Figure 10 | Voltage output performance of the BPCL *in vitro*........ 17

Supplementary Figure 11 | Stability characterization of the PEG........ 18

Supplementary Figure 12 | The standards of the injury level and the opacity score 19

Supplementary Figure 13 | Output performance and experimental setup for mice. 20

Supplementary Figure 14 | Corneal repair rate calculation for mouse and rabbit models. ... 21

Supplementary Figure 15 | Mice corneal alkali burn repair 22

Supplementary Figure 16 | Histological characterization of mouse 23

Supplementary Figure 17 | Mouse corneal repair with different parameters 24

Supplementary Figure 18 | Rabbit corneal alkali burn repair........ 26

Supplementary Figure 19 | Rabbit corneal alkali burn repair in the RB group 27

Supplementary Figure 20 | Histological characterization of rabbit 28

Supplementary Figure 21 | Corneal repair under endogenous EF and applied EF........ 29

Supplementary Figure 22 | Mouse and rabbit IFC images of multiple growth factors 30

Supplementary Figure 23 | Mouse and rabbit IHC staining images of VEGF 31

Supplementary Notes

Supplementary Note 1 | The cellular layers of the corneal epithelium. The corneal epithelium, the cornea's most anterior layer, consists of three cell types in 3-7 layers[1,2] (**Supplementary Figure 12a**). The morphology of epithelial cells differs amongst the three layers, which are the superficial layer, the wing cell layer, and the basal cell layer, from anterior to posterior. The superficial layer consists of flattened cells with apical microvilli. Beneath this is a layer of several wing cells. The basal layer is a single layer of columnar cells. Only these basal cells undergo mitosis and then migrate upwards, transforming into wing cells and, subsequently, into squamous cells. These cells are firmly adhered to their basement membrane by hemidesmosomes. During normal metabolism or injury, the epithelial surface cells of the cornea are constantly being sloughed away (continuous renewal every 4 to 7 days). Thus, the epithelium maintains a population of slow-cycling stem cells to replenish the tissue continually. In the grade II burn model, the corneal epithelial cells were partially missing, and the superficial stromal fibers were irregularly arranged, loose, and structurally disordered.

Supplementary Note 2 | The corneal repair rate calculation. During corneal injury repair, the relationship between S_I (the actual injury area) and the observed injury area (S_O) on cross-sectional mouse and rabbit eyeballs is shown in **Supplementary Figure 14b**. The injury area is a spherical cap located on the spherical surface of the animals' corneas. In geometry, the curved cap is a portion of the corneal sphere cut off by a plane. The relationship between the S_I and the S_O can be analyzed by the following Equations (1-4):

$$S_I = 2\pi R(R - h) = 2\pi R^2(1 - sin\theta) \quad (1)$$

$$S_O = \pi r^2 = \pi R^2 \cos^2\theta = \pi R^2(1 - \sin^2\theta) \quad (2)$$

$$r = Rcos\theta \quad (3)$$

$$\frac{S_I}{S_O} = \frac{2\pi R^2(1 - sin\theta)}{\pi R^2(1 - \sin^2\theta)} = \frac{2}{1 + sin\theta} \quad (4)$$

where R is the radius of the corneal sphere, r is the radius of the observed injury area (the base of the spherical cap), h is the height from the corneal sphere center to the cap center, and θ is the

polar angle from the horizontal rays from the center of the corneal sphere and the edge of the disk forming the base of the cap. The corneal radii are 1.414 mm and 7.26 mm for mice (R_M) and rabbits (R_R), respectively[3,4]. The observed injury areas of mice (S_{OM}) and rabbits (S_{OR}) were evaluated with Image J. The actual injury areas of mice (S_{IM}) and rabbits (S_{IR}) could be calculated by Equation (5):

$$S_I = \frac{2S_O}{1 + sin\theta} = \frac{2S_O}{1 + \sqrt{1 - \frac{S_O}{\pi R^2}}} \quad (5)$$

Supplementary Note 3 | Mouse cornea repair with different intervention parameters. Three other experimental parameters ((0.3 Hz, 1h), (0.3 Hz, 2h), and (1 Hz, 2h)) were selected to investigate the repair effect in the mouse model (**Supplementary Figure 17**). All groups were subjected to the same corneal injury surgery procedure (n = 4). The corneal injury was scored and documented once every 2 days after surgery and before euthanasia (**Supplementary Figure 17a**, left). These images demonstrate that three additional intervention groups had a similar repair process with the initial intervention group (1 Hz, 1h). In contrast, the MB group showed a delayed healing effect. The corneal epithelial defect healed rapidly, and corneal opacity was significantly relieved from day 0 to day 4 under the BPCL intervention. The injured cornea was basically repaired on day 6, marked by the burn trace blurring and almost corneal transparent, along with the opacity score approaching zero. Corneas at the injury site were collected on day 6 post-intervention for histological examination by H&E staining (**Supplementary Figure 17a**, right). Then we counted corneal clarity, repair rate, and epithelial thickness to analyze the repair effect of different parameters. The quantified opacity score over time is shown in **Supplementary Figure 17b**, opacity scores of the (1 Hz, 1h) and the (1 Hz, 2h) groups were slightly smaller than the three other groups ((0.3 Hz, 1h), (0.3 Hz, 2h), and MB) at day 2. From day 4, the gap of opacity score between four intervention groups and MB group was gradually widening, and there was no substantial difference for all intervention groups. The final opacity score for different groups is shown in **Supplementary Figure 17c**. The corneal clarity results revealed that the average score of the MB group was 2.25 ± 0.5, significantly higher than those of the intervention groups (0.75 ± 0.5 for (0.3 Hz, 1h), 0.5 ± 0.58 for (0.3 Hz, 2h), 0.4 ± 0.52 for (1.0 Hz, 1h), and 0.25 ± 0.5 for (1.0 Hz, 2h)). There was no statistically significant difference in opacity score among the four

intervention groups. For the repair rate, the corneal injury area under the BPCL intervention quickly decreased from day 0 to day 6, and the repair rate of the intervention group is much higher than that of the MB group (**Supplementary Figure 17d**). As shown in **Supplementary Figure 17e**, the repair rate in the MB group on day 6 was -71.4 ± 12.7%, significantly smaller than those of intervention groups (83.1 ± 4.4% for (0.3 Hz, 1h), 93.4 ± 7.3% for (0.3 Hz, 2h), 96.0 ± 3.4% for (1.0 Hz, 1h), and 97.8 ± 1.0% for (1.0 Hz, 2h)). H&E staining analysis confirmed that the intervention groups achieved effective re-epithelialization at the injury sites, whereas the MB group exhibited hypo-epithelialization with a smaller epithelial thickness (**Supplementary Figure 17a**, right). The epithelial thickness on day 6 was 19.33 ± 2.54 μm for the MB group, which was significantly smaller than those of the intervention groups (34.48 ± 2.98 μm for (0.3 Hz, 1h), 36.65 ± 0.93 μm for (0.3 Hz, 2h), 36.84 ± 2.56 μm for (1.0 Hz, 1h), and 37.65 ± 1.11 μm for (1.0 Hz, 2h)) (**Supplementary Figure 17f**). Similarly, there was no statistically significant difference in repair rate and epithelial thickness among the four intervention groups. Although the repair effect tends to improve with the increase of intervention time and frequency, there was no significant difference in corneal clarity, repair rate, and epithelial thickness for different intervention parameters ((0.3 Hz, 1h), (0.3 Hz, 2h), (1 Hz, 1h) and (1 Hz, 2h)).

Supplementary Figures

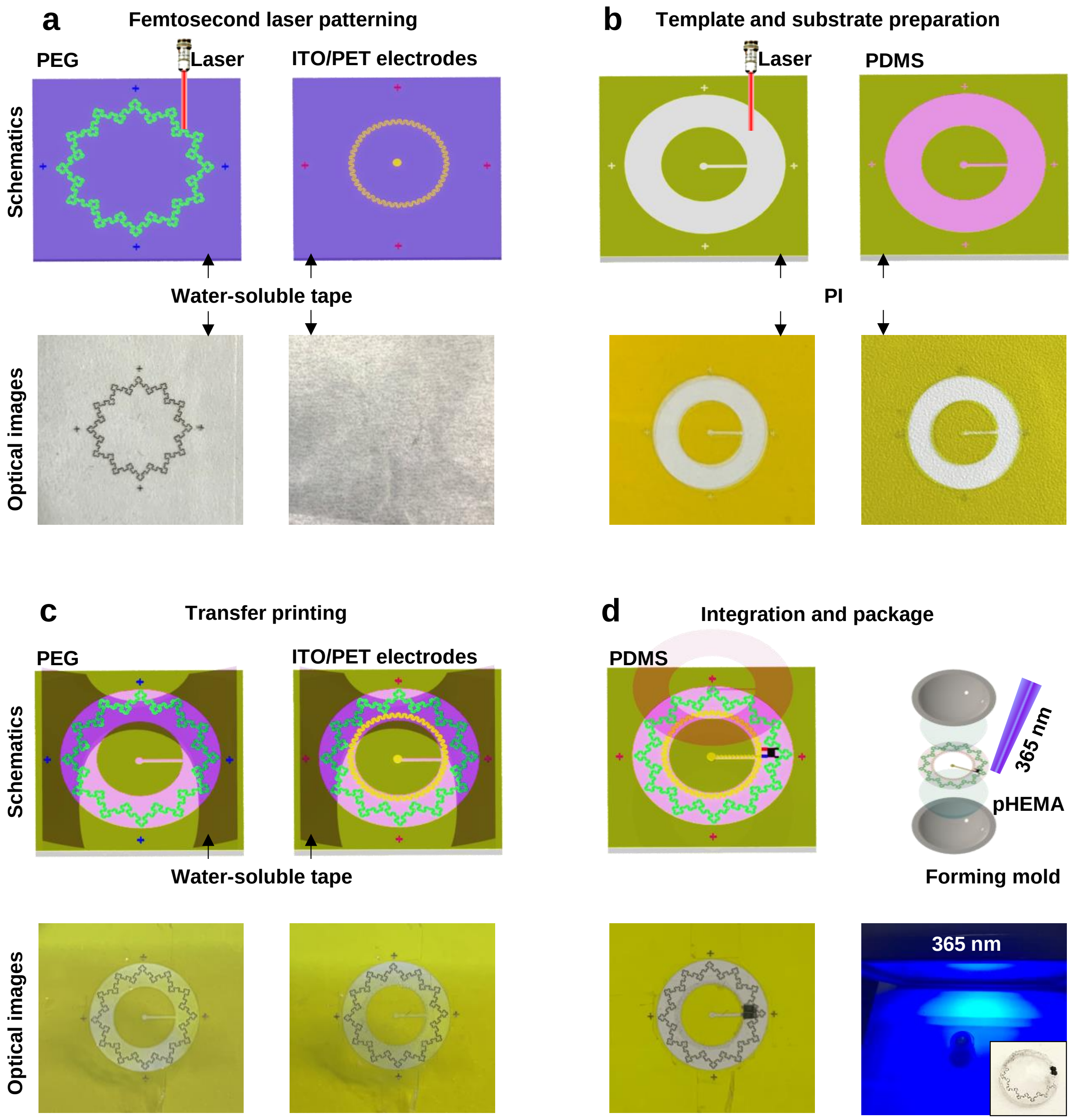

Supplementary Figure 1 | The fabrication procedure of the BPCL. (a) The piezoelectric polypropylene electret film with double-sided aluminum (Al) electrodes and ITO film sputtered on the PET substrate were prepared. Then, Al/electret/Al and ITO/PET films adhered to the water-soluble tape. The Al/electret/Al film and ITO/PET film were patterned to dendritic fractal star polygon and plate-like serpentine structures by femtosecond laser cutting technology, respectively.

(b) the polyimide (PI) film on a glass slide was also patterned by laser cutting to fabricate a template. Then PDMS solution was spin-coated on the template, acting as the bottom hydrophobic encapsulation layer of the PEG. **(c)** The piezoelectric film and concentric circular ITO electrodes were successively adhered to the template-based PDMS substrate by transfer printing technique according to bilayer alignment marks, followed by the water-soluble tape removed. **(d)** the ITO electrodes and the PEG component were connected *via* the micro rectifier with PDMS encapsulation. Then the peeled device was packaged by hydrophilic pHEMA in a mold with 365 nm light irradiation[5]. Abbreviations: PEG, polypropylene electret film generator; ITO, indium tin oxide; PET, polyethylene terephthalate; PDMS, polydimethylsiloxane; PI, polyimide; pHEMA, poly 2-hydroxyethyl methacrylate.

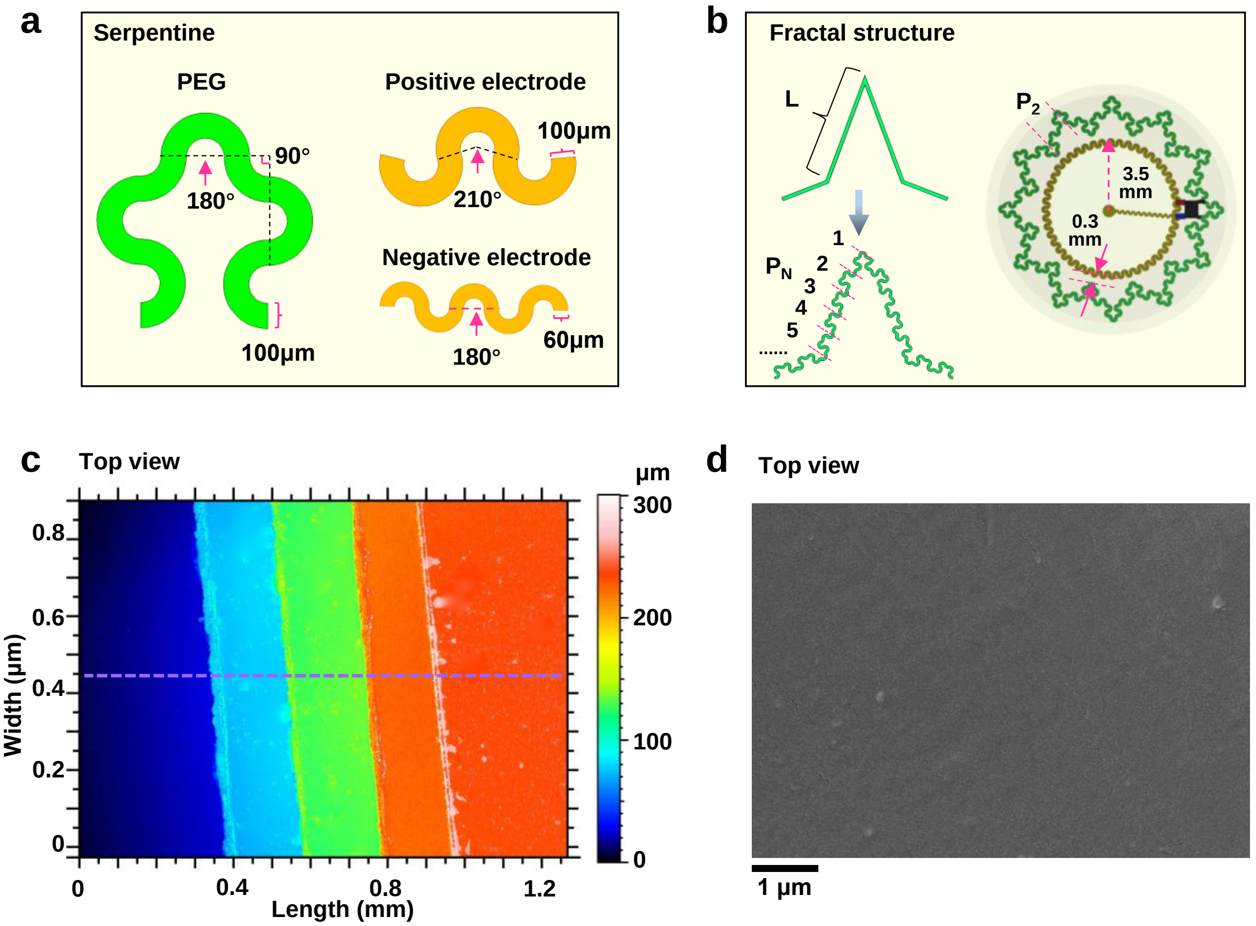

Supplementary Figure 2 | Characterization of the BPCL. (a) Serpentine geometry of the device components. The linewidth of the PEG and the positive ITO electrode was 100 μm, and the linewidth of the negative ITO was 60 μm. **(b)** Parametric characterization of the partially enlarged fractal structure and the overall PEG. The side length of the star polygon was defined as *L*, which was replaced by fractal structures with different numbers of periods (P_N). The P_N was 2 when the D_n was set to be 12. Concentric circular ITO electrodes (radius~3.5 mm) were deposited 0.3 mm apart from the PEG component and connected to the PEG *via* the micro rectifier. **(c)** Top-view three-dimensional microscope images of the multilayer structures. **(d)** Top-view SEM image of the uniform piezoelectric electret film. Abbreviations: PEG, polypropylene electret film generator; *L*, the side length of the star polygon; P_N, number of fractal periods.

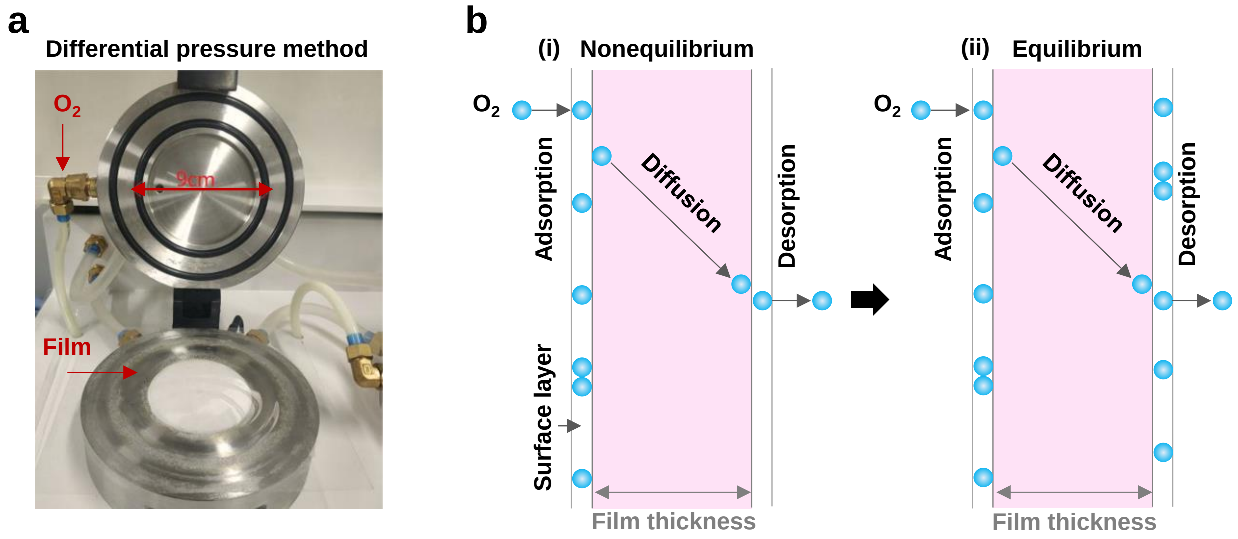

Supplementary Figure 3 | Oxygen permeability characterization of encapsulation layers. (a) Image of the gas permeability test system based on the differential pressure method[6,7]. **(b)** Process of oxygen permeation through the thin film.

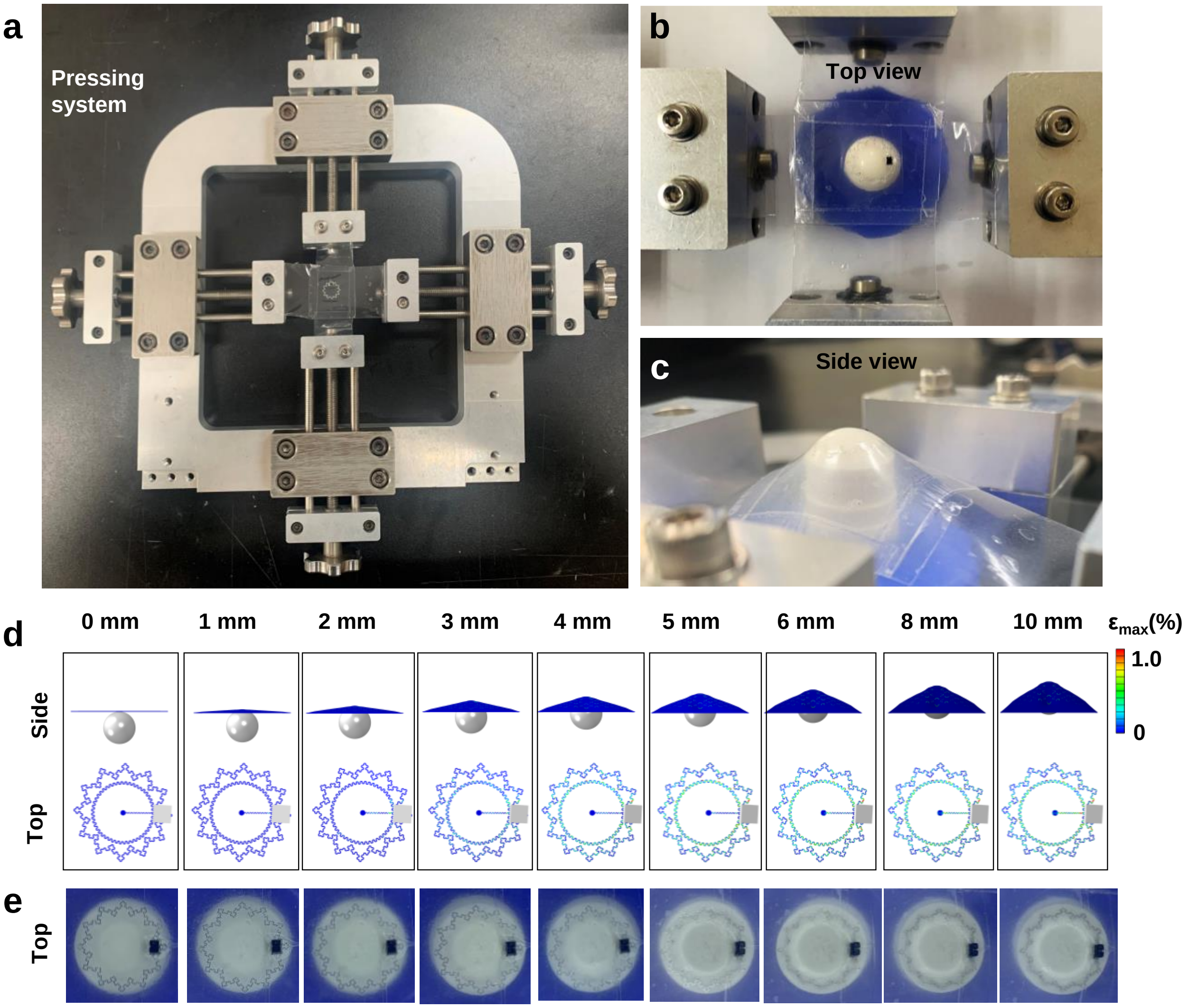

Supplementary Figure 4 | Mechanical robustness of the BPCL. (a) Optical image of the overall pressing system. **(b)** Top view and **(c)** Side view of the BPCL pressed with a spherical plastic ball with a radius of 6 mm. **(d)** FEA results of the BPCL under a series of pressing heights (from 0 mm to 10 mm) demonstrated that the strain (≤ 1.0%) was evenly distributed on the fractal structure and serpentine lines. **(e)** Corresponding height experimental results of the BPCL. The BPCL can be repeatedly pressed up to 10 mm without damage.

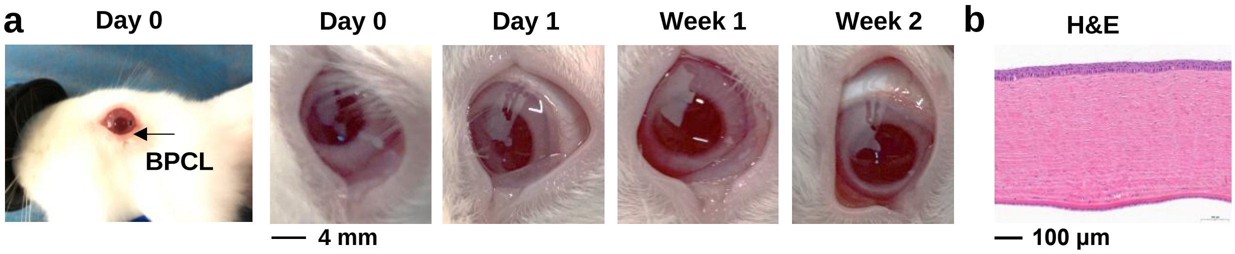

Supplementary Figure 5 | *In vivo* biocompatibility verification of the BPCL. (a) A series of images on the rabbit cornea over time. The water- and oxygen-permeable BPCL was worn on the rabbit for two weeks. The rabbit cornea was observed and photographed on day 0, day 1, week 1, and week 2, and the cornea preserved transparency during the observation period. **(b)** H&E staining images of the cornea on week 2. On week 2 post-observation, corneal tissue at the injury sites was collected for histological examination by H&E staining. Histological analysis revealed that the epithelium of the cornea wearing BPCL was complete and uniform, presenting intact epithelium tightly connected to the underlying stromal tissue. These results confirmed the encapsulated BPCL is biocompatible in vivo. Abbreviations: BPCL, blink-driven piezoelectric contact lens; H&E, hematoxylin-eosin.

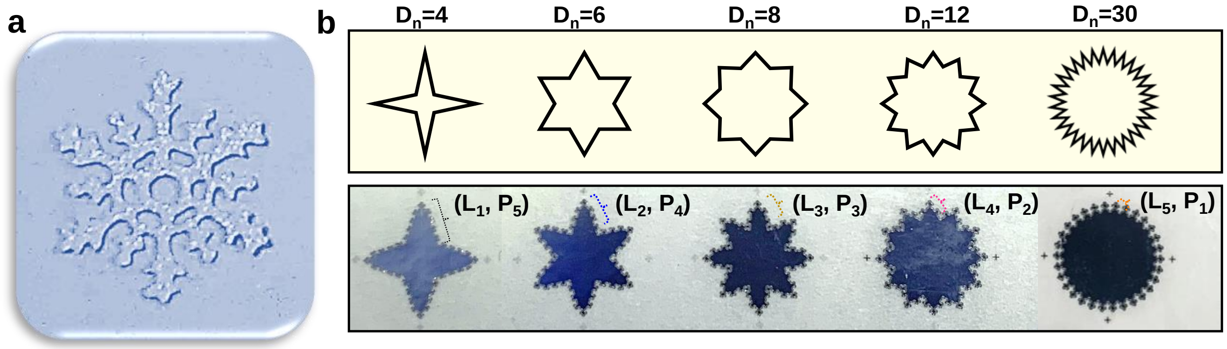

Supplementary Figure 6 | Fractal star polygon structure of the snowflake-inspired BPCL. (a) Schematic of a natural snowflake. **(b)** P_N was 5, 4, 3, 2, and 1 when the D_n (the number of dendrites) was designed to be 4, 6, 8, 12, and 30 respectively (Supplementary Figure 2). The corresponding piezoelectric areas were 6.1 mm^2, 7.8 mm^2, 8.4 mm^2, 9.4, and 10.6 mm^2. Abbreviations: D_n, dendrite number; L, the side length of the star polygon; P_N, number of fractal periods.

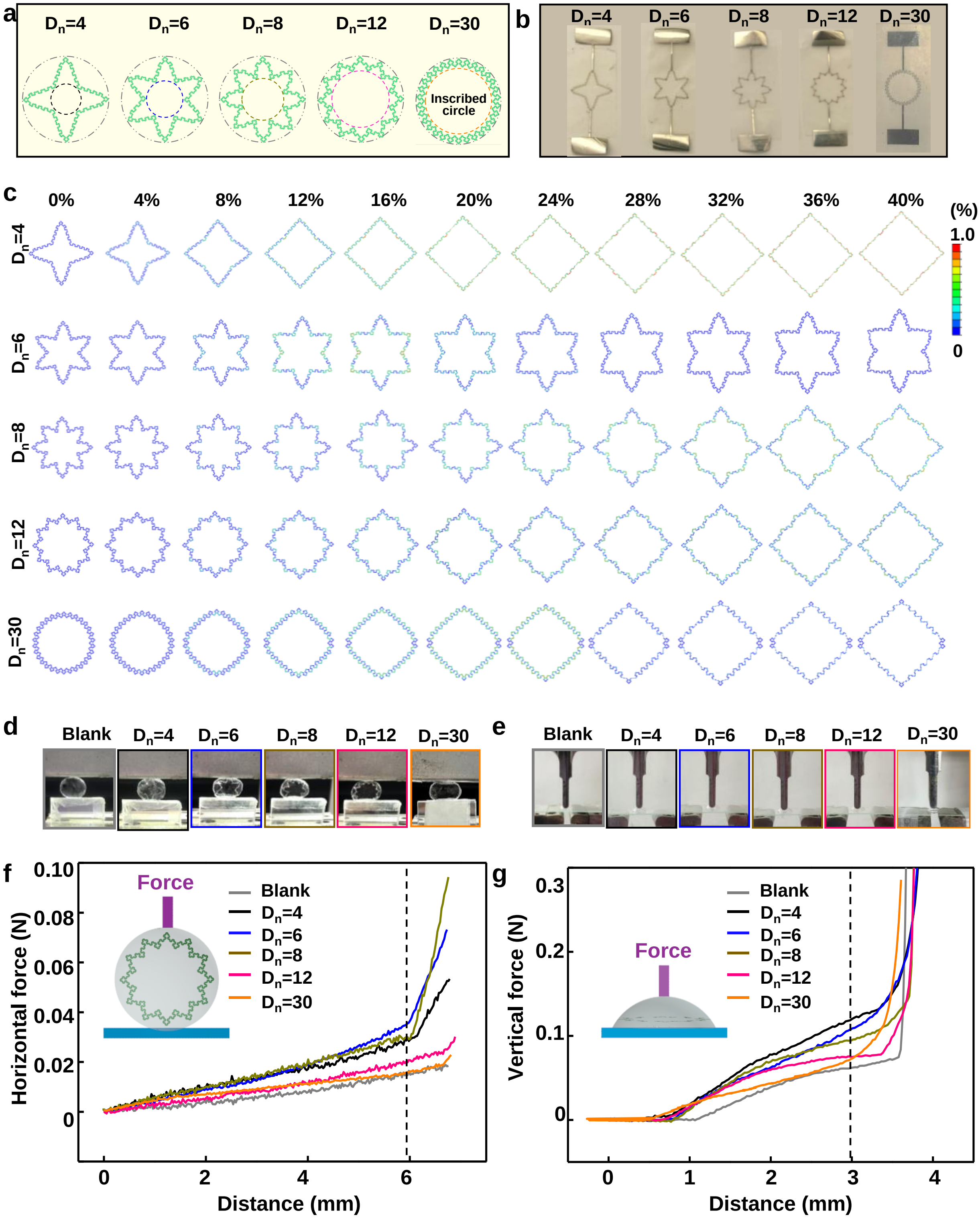

Supplementary Figure 7 | Performance of the PEGs with a different number of dendrites. (a) The inscribed circle area was 15.9 mm^2, 23.0 mm^2, 29.2 mm^2, 52.1 mm^2, and 72.9 mm^2 when the D_n was 4, 6, 8, 12, and 30, respectively. **(b)** Piezoelectric electrets with a different D_n for voltage

output measurement. Pattern design at both ends facilitated testing. **(c)** FEA results of the PEGs under biaxial tensile strain (0%-40%) demonstrate that the strain subjected by the PEG (≤1%) was evenly distributed on the fractal structure. The stretchability was defined as the critical point at which 1% strain appeared on the PEG. The defined stretchability was 16%, 16%, 28%, 36%, and 40% when the D_n was 4, 6, 8, 12, and 30, respectively. **(d)** Optical image of the experimental setup for three-point bending test with the device positioned vertically. **(e)** Optical image of the experimental setup for three-point bending test with the device positioned horizontally. **(f)** The horizontal flexural force versus deformation with the device positioned vertically. **(g)** The vertical flexural force versus deformation with the device positioned horizontally. The horizontal and vertical flexibility of the PEG-based BPCL was defined as the reciprocal of horizontal (6 mm) and vertical (3 mm) flexural force, respectively. The (vertical flexibility, horizontal flexibility) values were (8.3, 34.5), (9.2, 27.0), (10.4, 34.5), (13.5, 50.0), and (13.7, 62.5) when the D_n was 4, 6, 8, 12, and 30, respectively. Abbreviations: D_n, dendrite number.

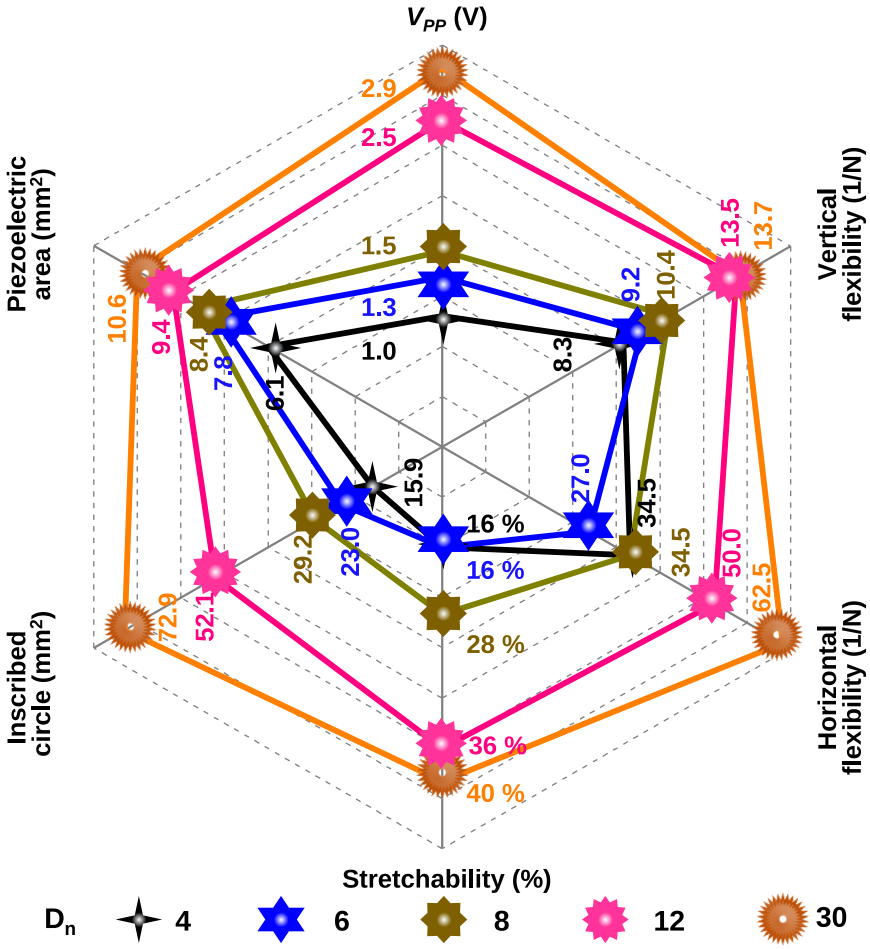

Supplementary Figure 8 | Radar chart of different PEGs for performance comparison. Compared with other geometric parameters (D_n = 4, 6, and 8), PEGs (D_n = 12 and D_n = 30) had optimized overall performance under the evaluation criteria of the piezoelectric area, inscribed circle area, voltage output in plane condition, stretchability, and mechanical properties. Abbreviations: V_{pp}, peak-to-peak voltage; D_n, dendrite number.

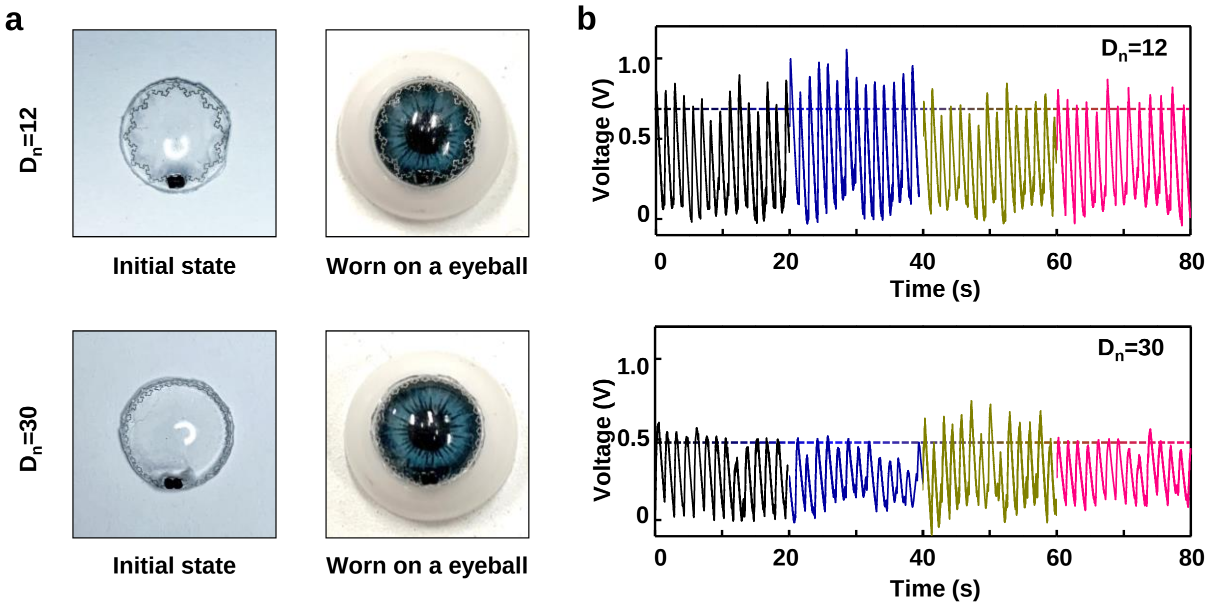

Supplementary Figure 9 | Performance comparison of the BPCLs (*D_n* =12 and 30). (a) Optical images of BPCLs (D_n =12 and 30) in initial state and worn on an eyeball. **(b)** Voltage output of BPCLs (D_n =12 and 30) during blink *in vivo*. The average voltage output of the BPCL with D_n =30 was ~0.5 V, significantly smaller than that of BPCL (D_n =12). This phenomenon could be attributed to that most of the piezoelectric material of PEG (D_n = 30) is located at the BPCL edge, and the piezoelectric activation area may be relatively smaller during the blink process. This comparison confirmed that the BPCL (D_n =12) could convert spontaneous daily blink motions into stable electric pulses more effectively. Thus, the PEG (D_n = 12) was chosen for further animal studies. Abbreviations: D_n, dendrite number.

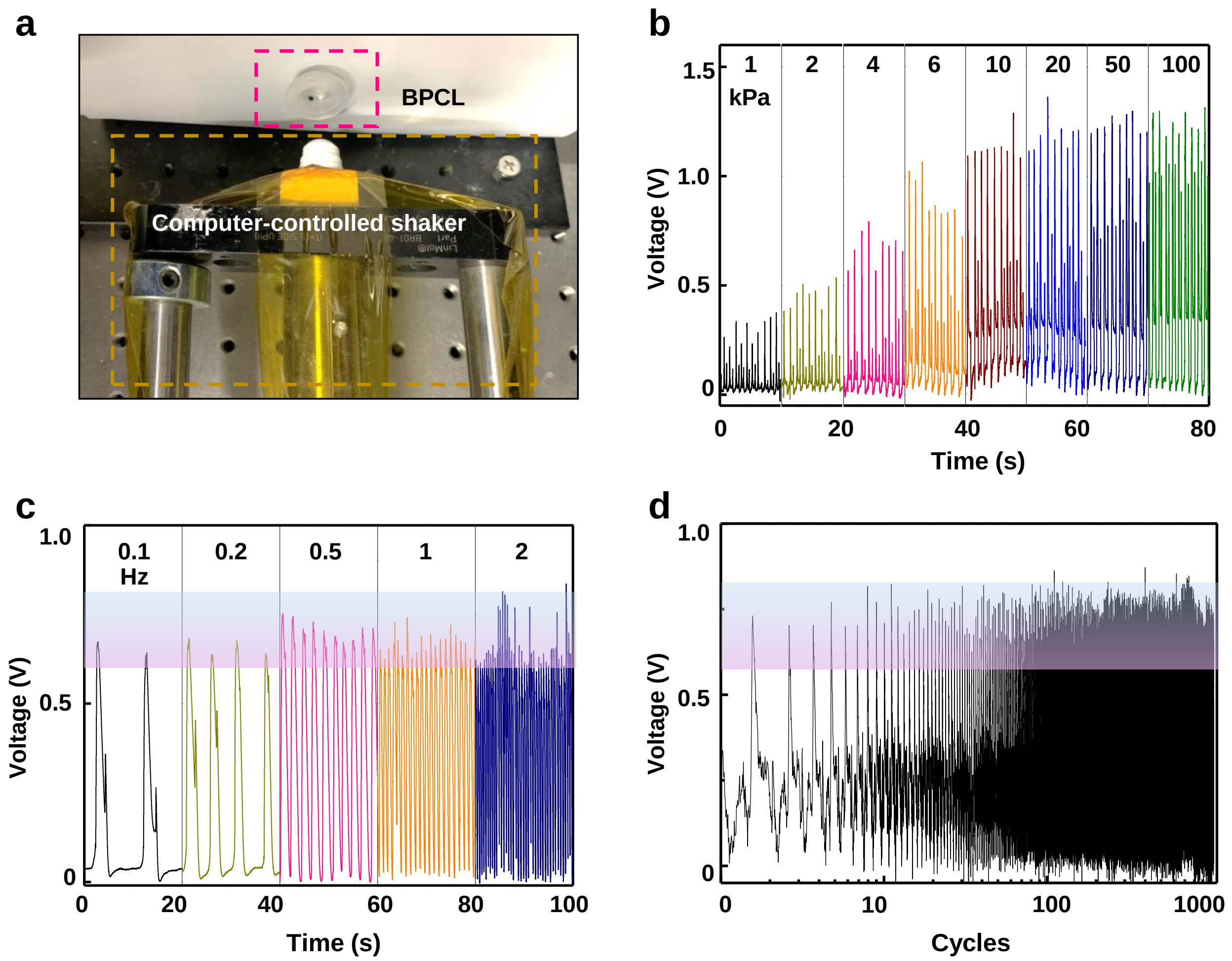

Supplementary Figure 10 | Voltage output performance of the BPCL *in vitro*. (a) The BPCL was driven by a computer-controlled shaker. The pressure was calibrated with a commercial pressure transducer. **(b)** The voltage output performance of the BPCL under different pressure. The output voltage increased as the pressure gradually increased and then remained constant. **(c)** The voltage output (~ 4kPa) performance of the BPCL with different frequencies. **(d)** Long-term stability test of the BPCL for over 1000 cycles. Abbreviations: BPCL, blink-driven piezoelectric contact lens.

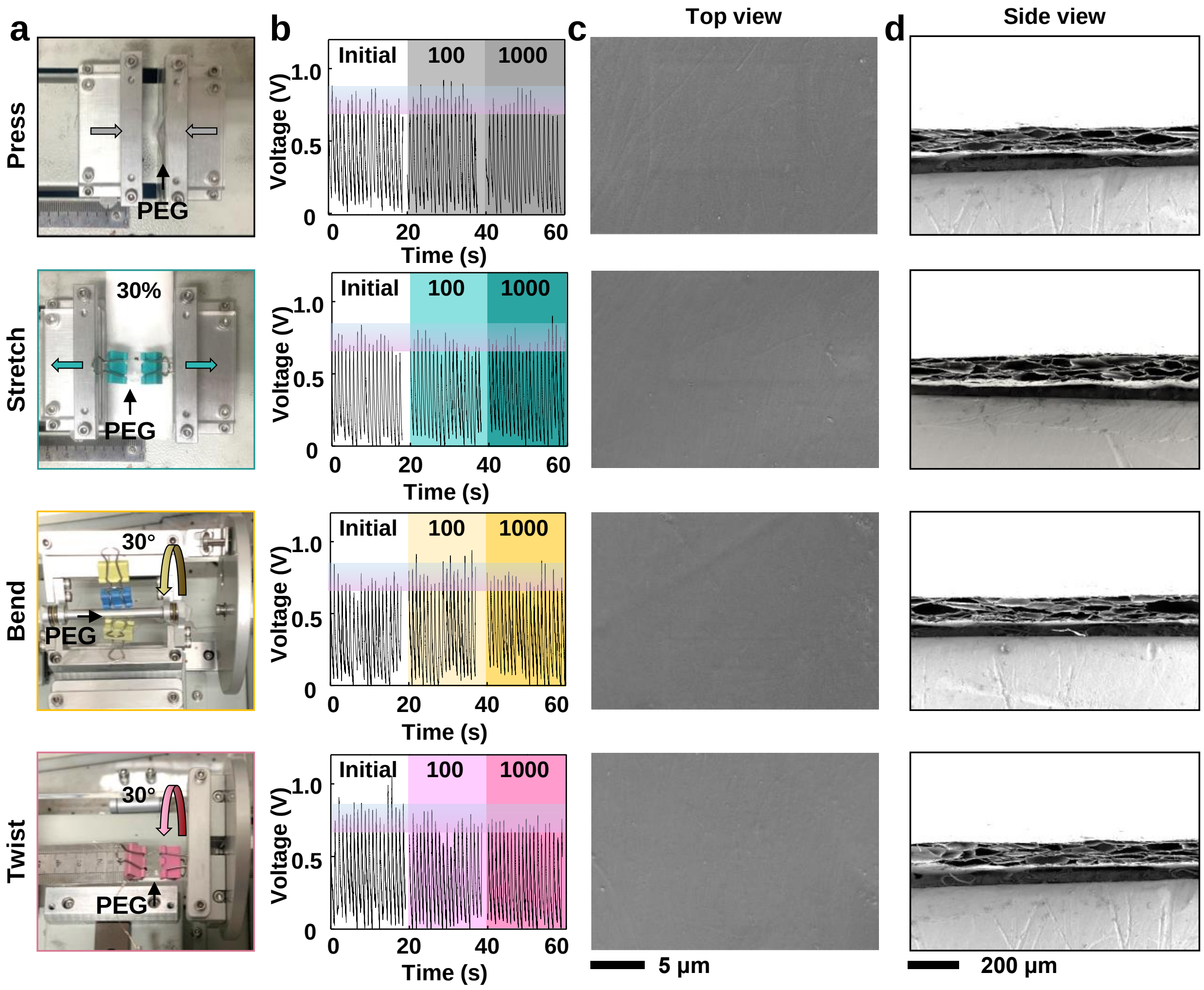

Supplementary Figure 11 | Stability characterization of the PEG structure and performance. **(a)** Optical images of PEGs being pressed, stretched, bent and twisted. The range of compression distance, tensile strain, bending angle, and twisting angle were (0.5 mm -2 mm), (0% - 30%), (0° - 30°), and (0° - 30°), respectively. **(b)** Corresponding long-term voltage stability characterization of the PEGs. The nearly unchanged voltage amplitude (~0.7 V) confirmed the excellent robustness and durability of the PEGs after 0 (initial), 100, and 1000 deformation cycles. **(c)** Top-view SEM images showed all the PEG surface was flat and without obvious cracks. **(d)** Side-view SEM images displayed that the porous loose structure is complete and without cross-link broken. These output voltage and microstructure results revealed that PEG has good structural integrity without any visible defects after long-term operation. Abbreviations: PEG, polypropylene electret film generator.

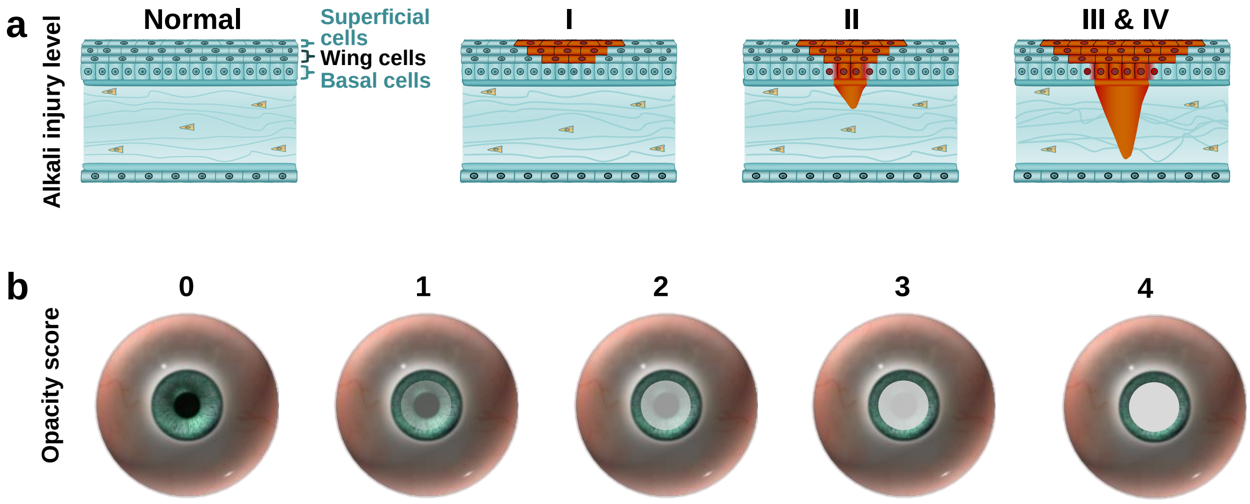

Supplementary Figure 12 | The standards of the corneal injury level and the opacity score[8-12]. **(a)** Corneal injury level standard: normal corneal structure is complete, the epithelial cell layer is flat, and the stromal layer fibers are neatly arranged. The cellular layers of the corneal epithelium is described in Supplementary Note 1. In mild (grade I) burns, the corneal epithelial layer is incomplete, but the fibers of the stromal layer are neatly arranged and structurally intact. In moderate (grade II) burns, the corneal epithelial layer is partially missing, and the superficial stromal fibers are irregularly arranged, loose, and structurally disordered. In contrast, the deep fibers are relatively neat, dense, and regular. In severe (grade III and IV) burns, the corneal epithelium is missing, and the full-thickness fibers of the stroma are irregularly arranged, loose, and structurally disordered. Severe burns may cause corneal ulcers or perforations. **(b)** The criteria for corneal opacity (N = 0, 1, 2, 3, and 4) were as follows: score 0, totally clear, with no opacity seen by any method of slit-lamp microscopic examination; score 1, a haze of minimal density seen with difficulty with direct and diffuse illumination; score 2, mild haze easily visible; score 3, moderately dense opacity that partially obscured the iris details; and score 4, severely dense opacity that completely obscured the details of intraocular structures.

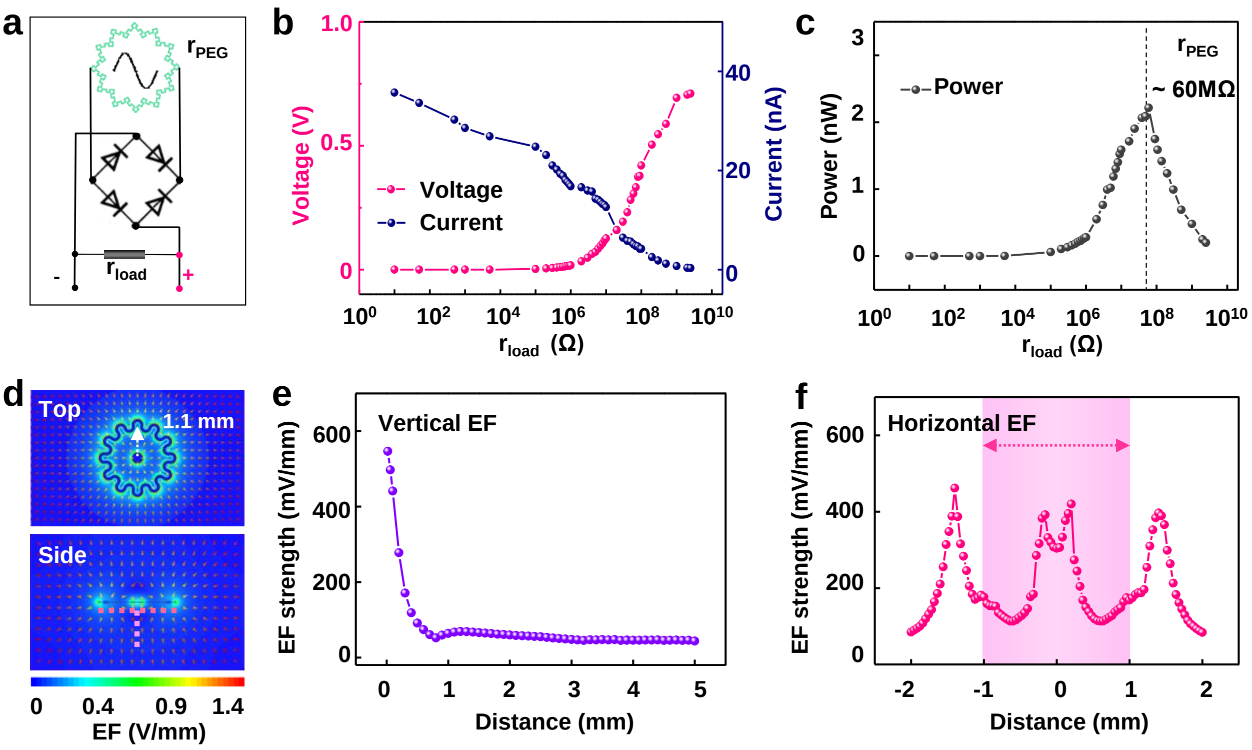

Supplementary Figure 13 | BPCL output performance and experimental setup for mice. **(a)** Schematics of the BPCL-based repair system for animal models. **(b)** Voltage and current output function of load resistance (r_{load}) at a pressure of 4kPa. **(c)** Output power as a function of load resistance calculated from the voltage and current of the BPCL. The internal resistance of the PEG (r_{PEG}) is about 60MΩ[13]. **(d)** Top-view and side-view AMFES simulated EF distribution inside a cornea. Voltage amplitude was adjusted to ~230 mV with an external resistor (30 MΩ). **(e)** Vertical and **(f)** Horizontal EF strength as a function of the depth and width extrapolated from Supplementary Figure 10d (bottom). The horizontal EF strength of the injury site at the 200 μm depth was in the approximate range of 120 mV/mm to 420 mV/mm. The corneal injury area was highlighted in pink. Abbreviations: PEG, polypropylene electret film generator; EF, electric field.

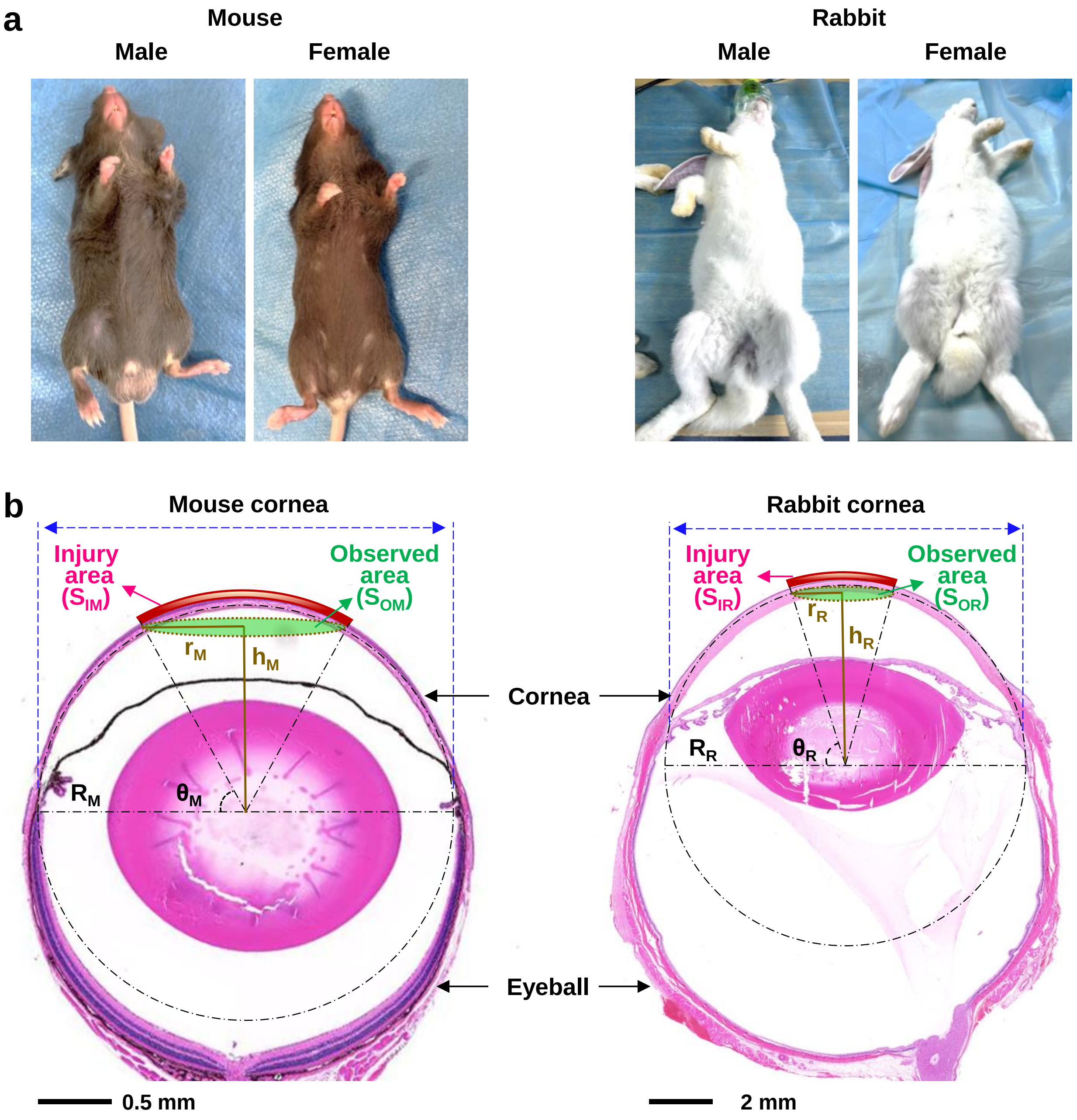

Supplementary Figure 14 | Corneal repair rate calculation for mouse and rabbit models. (a) Male and female animals to verify the repair effect. **(b)** Relationship between S_I and S_O. S_I, the actual injury area; S_O, the observed injury area; R, the radius of the corneal sphere; r, the radius of the observed injury area; h, the height from the corneal sphere center to the cap center; θ, the polar angle from the horizontal rays from the center of the corneal sphere and the edge of the disk forming the base of the cap.

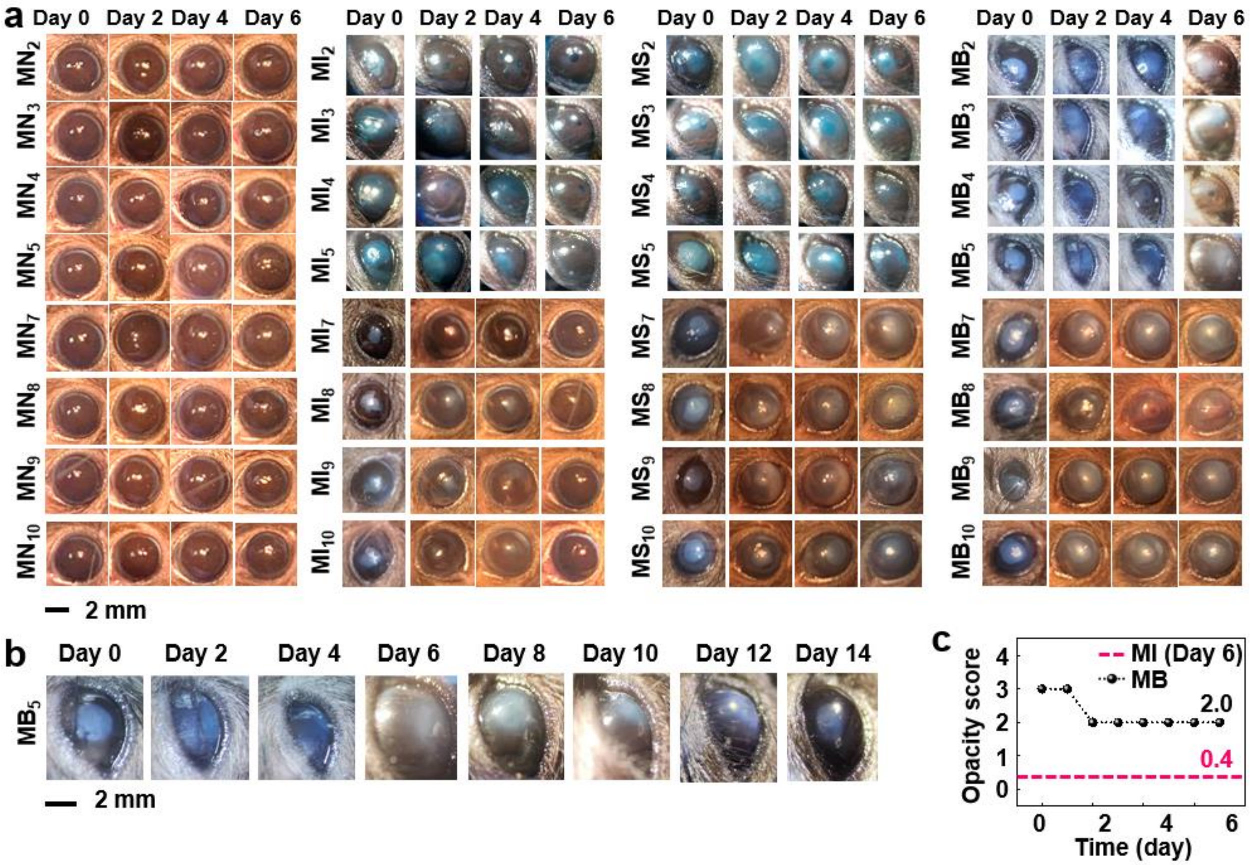

Supplementary Figure 15 | Mice corneal alkali burn repair during the BPCL intervention. **(a)** A series of images on the alkali burn cornea over time of the MN, MI, MS, and MB groups. **(b)** The burn trace did not completely disappear on day 14 without the BPCL intervention. **(c)** Corneal opacity score over time in the MB group. The pink dash line represents corneal opacity for MI group on day 6. Abbreviations: MN: normal mice without cornea injury; MI, mice in the intervention group; MS, mice in the sham group; MB, mice in the blank control group.

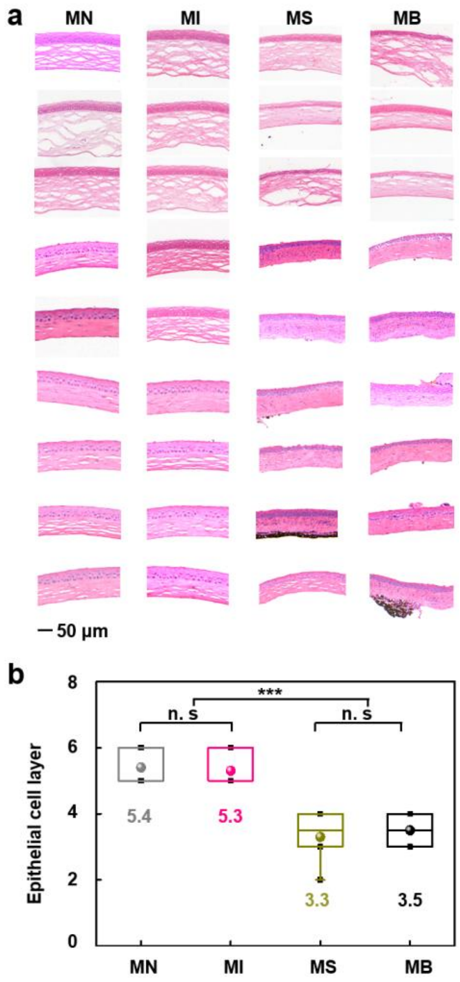

Supplementary Figure 16 | Histological characterization of mouse. (a) H&E staining images of post-intervention mouse corneas at the alkali burn site. **(b)** Comparison of epithelial cell layer for different mouse groups, P <3.5×10^{-6} for MN/MI and MS/MB, n=10 independent samples. The epithelial cell layer on day 6 were 5.4 ± 0.52 and 5.3 ± 0.48 for MN and MI groups, respectively, which were significantly bigger than those of the control groups (3.3 ± 0.82 for MS, and 3.5 ± 0.53 for MB). In box plots (b), the dot is the mean, the centerline is the median, box limits are the lower quartile (Q1) and upper quartile (Q3), and whiskers are the most extreme data points that are no more than 1.5 × (Q3 - Q1) from the box limits. Data were analyzed by parametric two-tailed Student's *t*-test, n.s and *** represent nonsignificant (P > 0.05) and P < 0.001, respectively. Differences were considered significant at P < 0.05. Abbreviations: MN: normal mice without cornea injury; MI, mice in the intervention group; MS, mice in the sham group; MB, mice in the blank control group.

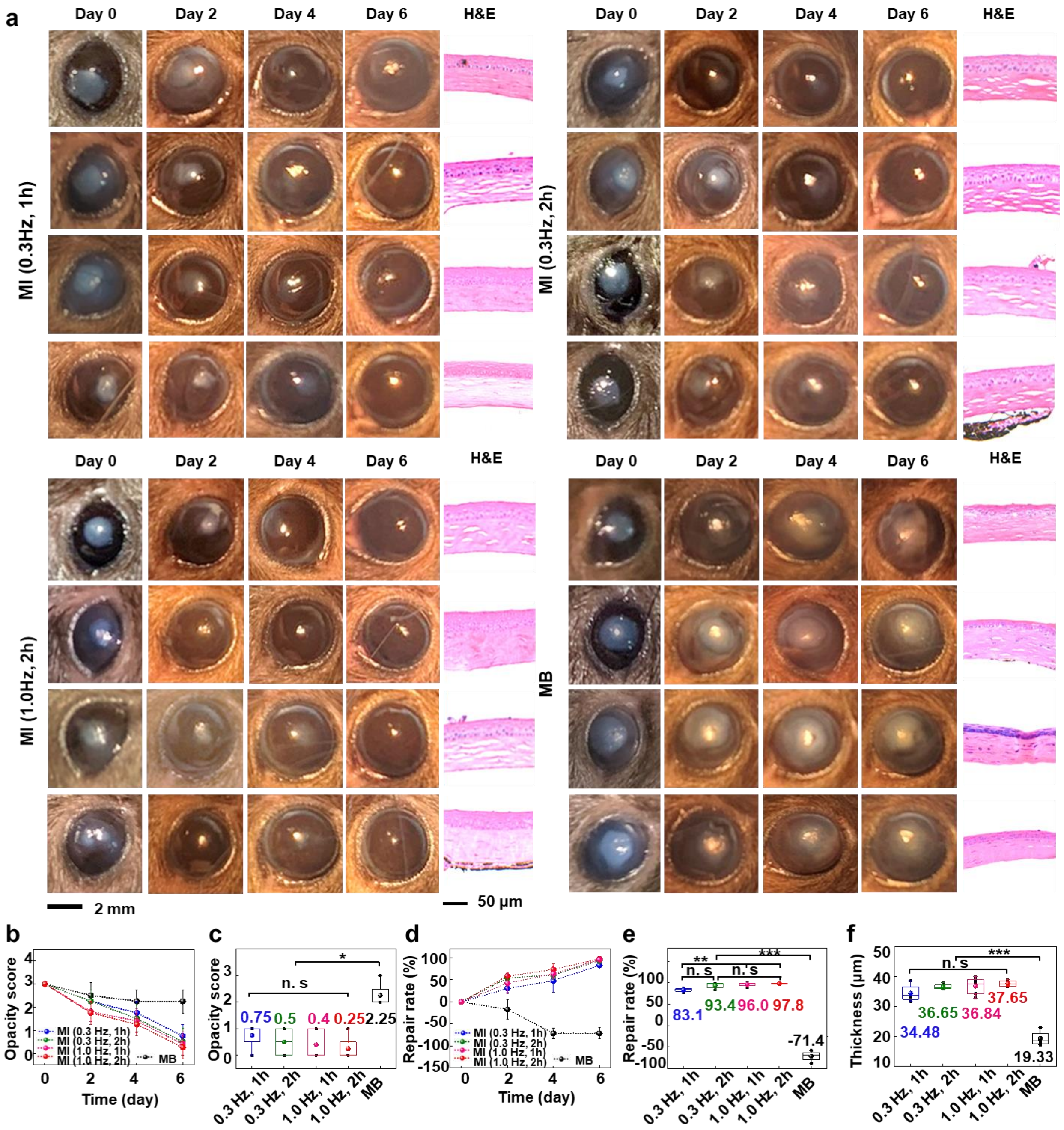

Supplementary Figure 17 | Mouse corneal repair with different parameters. (a) Images on the alkali burn cornea over time of the MI (0.3 Hz, 1h), MI (0.3 Hz, 2h), MI (1.0 Hz, 2h), and MB groups (n=4). **(b)** Corneal opacity score over time in different mouse groups. **(c)** Comparison of corneal opacity for different mouse groups on day 6, P <0.025 for MI groups and MB. **(d)** Corneal repair rate over time of the injury area. **(e)** Comparison of corneal repair rate on day 6 for different mouse groups, P =0.0056 for MI (0.5 Hz, 1h) and MI (1.0 Hz, 2h), P < 5×10^{-7} for MI groups and

MB, n=4 independent mice in Supplementary Figures 17b-e. **(f)** Comparison of epithelial thickness for different mouse groups, $P < 2.5\times10^{-4}$ for MI groups and MB, n=4 independent samples. All data in (b) and (d) are presented as means ± SD. In box plots (c, e, and f), the dot is the mean, the centerline is the median, box limits are the lower quartile (Q1) and upper quartile (Q3), and whiskers are the most extreme data points that are no more than 1.5 × (Q3 - Q1) from the box limits. Data were analyzed by non-parametric two-sided Mann-Whitney *U* test (c) and parametric two-tailed Student's *t*-test (e and f). n.s, *, and *** represent nonsignificant ($P > 0.05$), $P < 0.05$, and $P < 0.001$, respectively. Differences were considered significant at $P < 0.05$. Abbreviations: MI, mice in the intervention group; MB, mice in the blank control group.

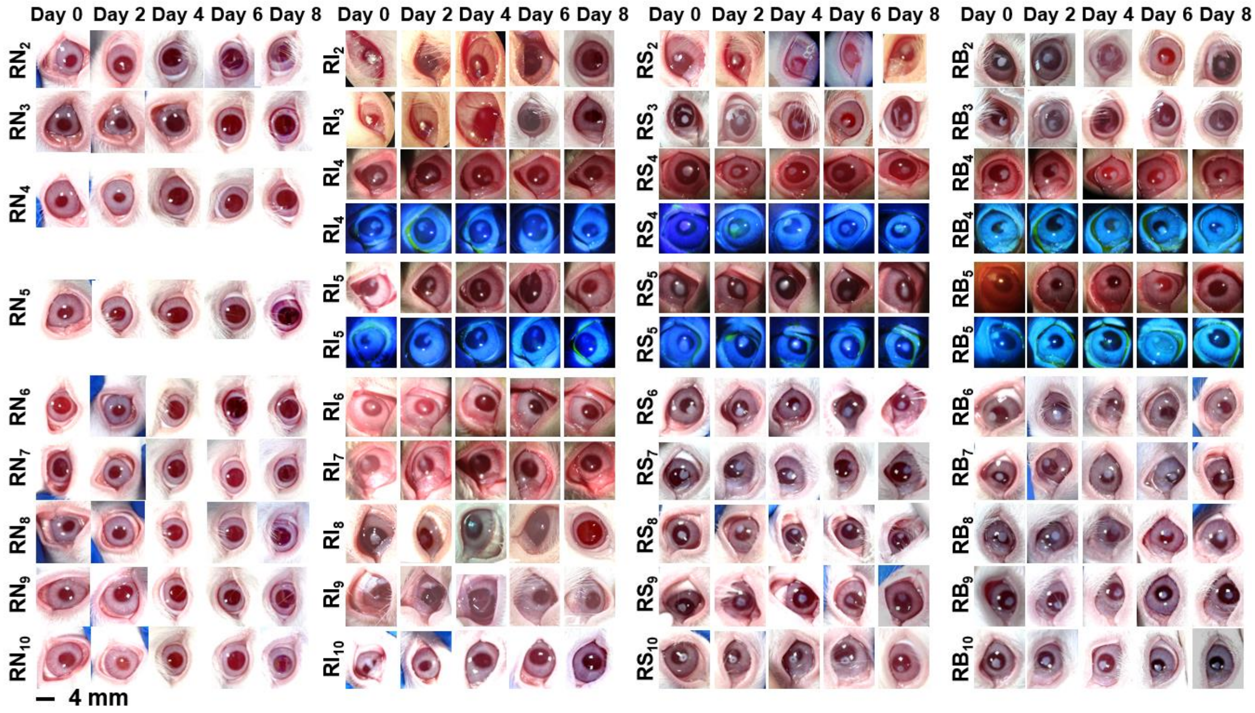

Supplementary Figure 18 | Rabbit corneal alkali burn repair during the BPCL intervention. A series of images on the alkali burn cornea over time of the RN, RI, RS, and RB groups. Abbreviations: RN: normal rabbits without cornea injury; RI, rabbits in the intervention group; RS, rabbits in the sham group; RB, rabbits in the blank control group.

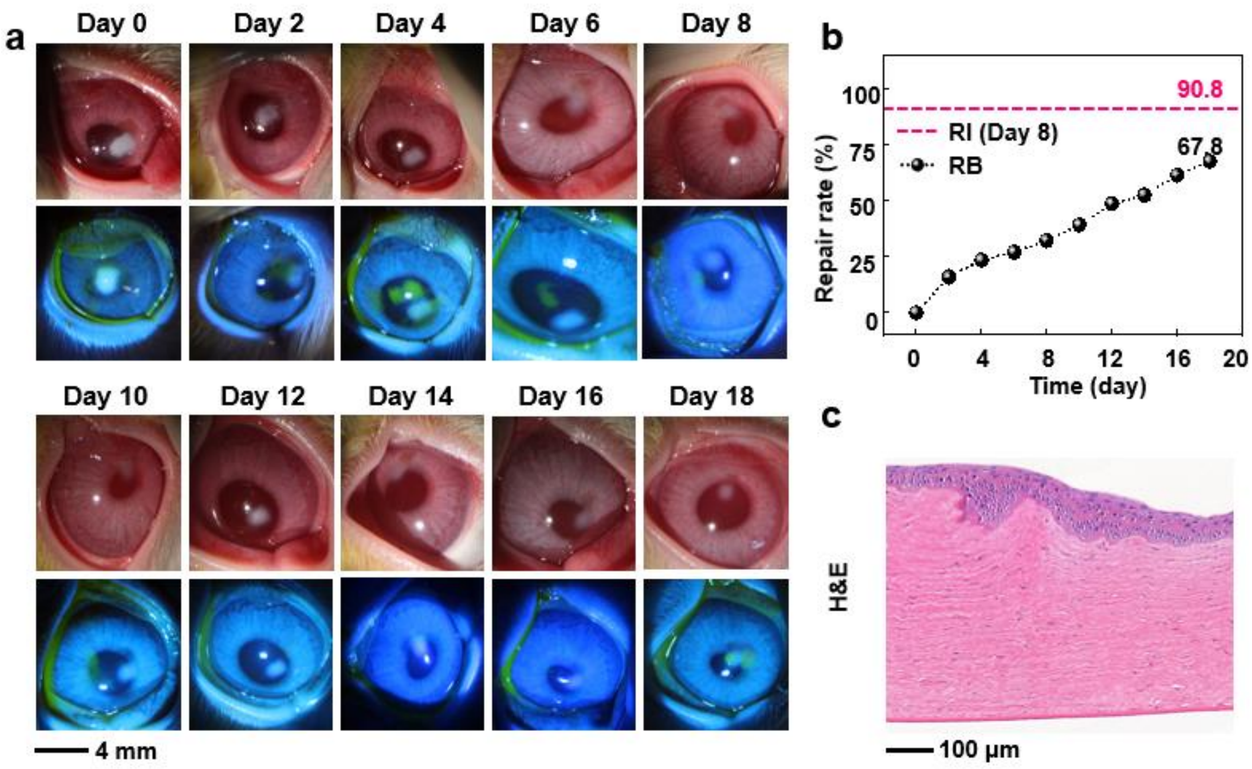

Supplementary Figure 19 | Rabbit corneal alkali burn repair in the RB group. (a) Corneal opacity score over time in the RB group. **(b)** Corneal repair rate over time of the injury area in the RB group. The pink dash line represents corneal opacity for RB group on day 8. **(c)** H&E staining images of the cornea with uneven thickness at the alkali burn site. Abbreviations: RI, rabbits in the intervention group; RB, rabbits in the blank control group; H&E, hematoxylin-eosin.

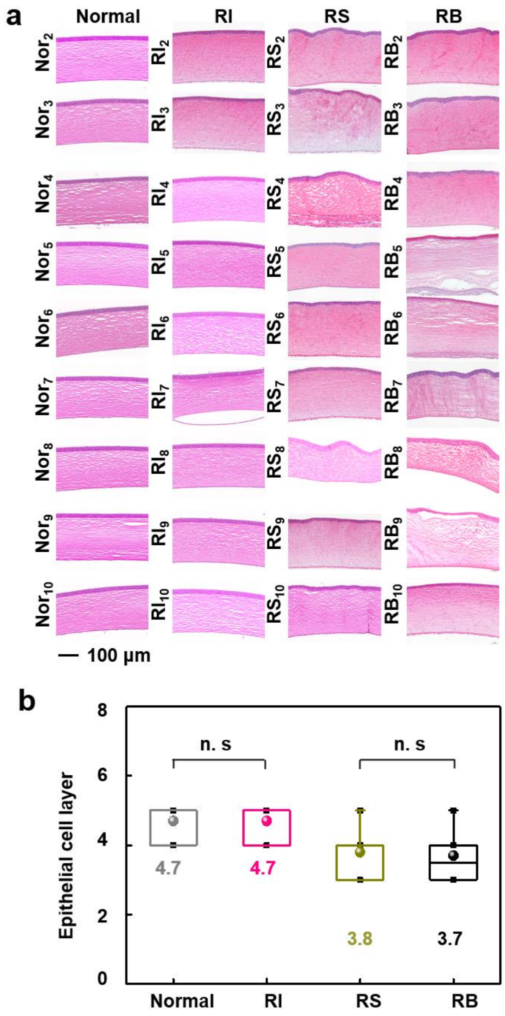

Supplementary Figure 20 | Histological characterization of rabbit. (a) H&E staining images of post-intervention rabbit corneas at the alkali burn site. **(b)** Comparison of epithelial cell layer for different rabbit groups (n=10 independent samples). The epithelial cell layer on day 8 were 4.7 ± 0.48, 4.7 ± 0.48, 3.8 ± 0.63, and 3.7 ± 0.82 for the RN, RI, RS, and RB groups, and there was no significant difference in epithelial cell layer among the four groups. In box plots (b), the dot is the mean, the centerline is the median, box limits are the lower quartile (Q1) and upper quartile (Q3), and whiskers are the most extreme data points that are no more than 1.5 × (Q3 - Q1) from the box limits. Data were analyzed by parametric two-tailed Student's *t*-test, n.s represents nonsignificant ($P > 0.05$). Differences were considered significant at $P < 0.05$. Abbreviations: RN: normal rabbits without cornea injury; RI, rabbits in the intervention group; RS, rabbits in the sham group; RB, rabbits in the blank control group.

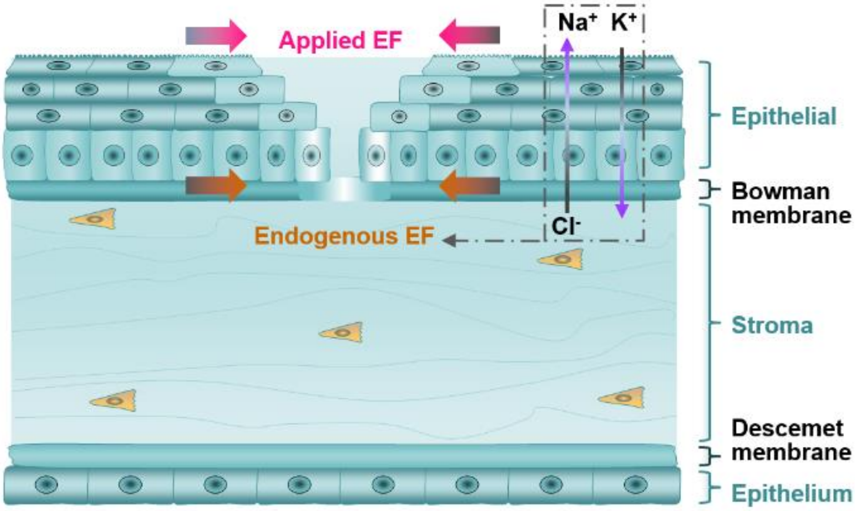

Supplementary Figure 21 | Corneal repair under endogenous EF and applied EF[14,15]. In the normal cornea, active transport of ions (Na^+, K^+, Cl^-) across the membrane generates internal negative and external positive membrane potentials named transepithelial potential (TEP). The disruption of a corneal epithelial layer and TEP induces the endogenous electric field (EF) from the edge to the center (brown arrow). The endogenous EF can guide the directional migration of epithelial cells to the injury center until the corneal repair process is completed. Applying EF in the default direction (pink arrow) will strengthen the injury-induced endogenous EF and accelerate the corneal injury repair. Abbreviations: EF, electric field.

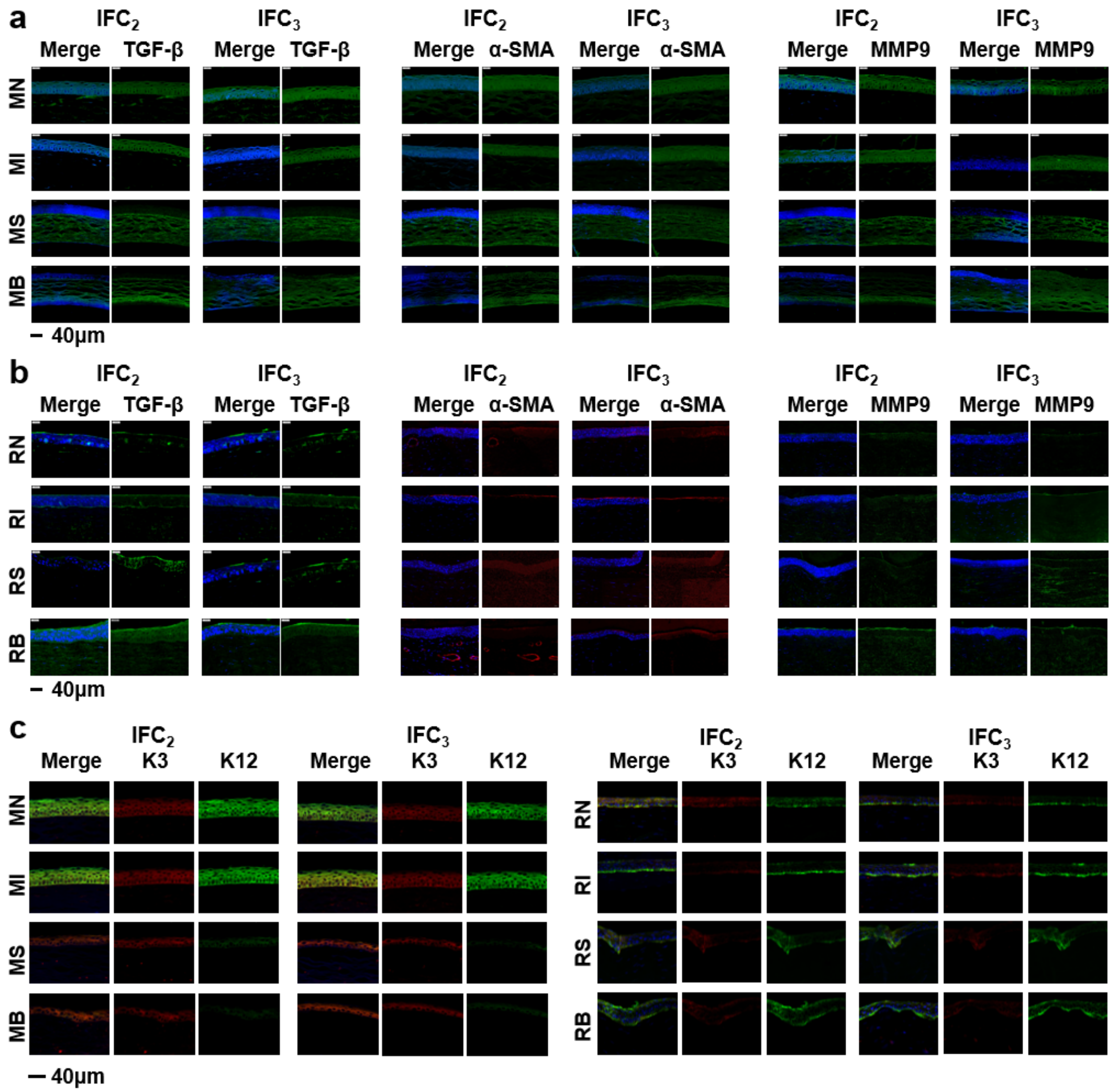

Supplementary Figure 22 | Mouse and rabbit IFC images of multiple growth factors. (a) Mouse IFC staining images of multiple growth factors, including TGF-β, α-SMA, and MMP9. **(b)** Rabbit IFC staining images of multiple growth factors, including TGF-β, α-SMA, and MMP9. **(c)** Mouse and rabbit IFC staining images of growth factors including K3 and K12. Abbreviations: MN: normal mice without cornea injury; MI, mice in the intervention group; MS, mice in the sham group; MB, mice in the blank control group; RN: normal rabbits without cornea injury; RI, rabbits in the intervention group; RS, rabbits in the sham group; RB, rabbits in the blank control group; IFC, immunofluorescence; TGF-β, transforming growth factor beta; α-SMA, alpha-smooth muscle actin; MMP9, matrix metalloproteinase 9; K3, Keratin 3; K12, Keratin 12.

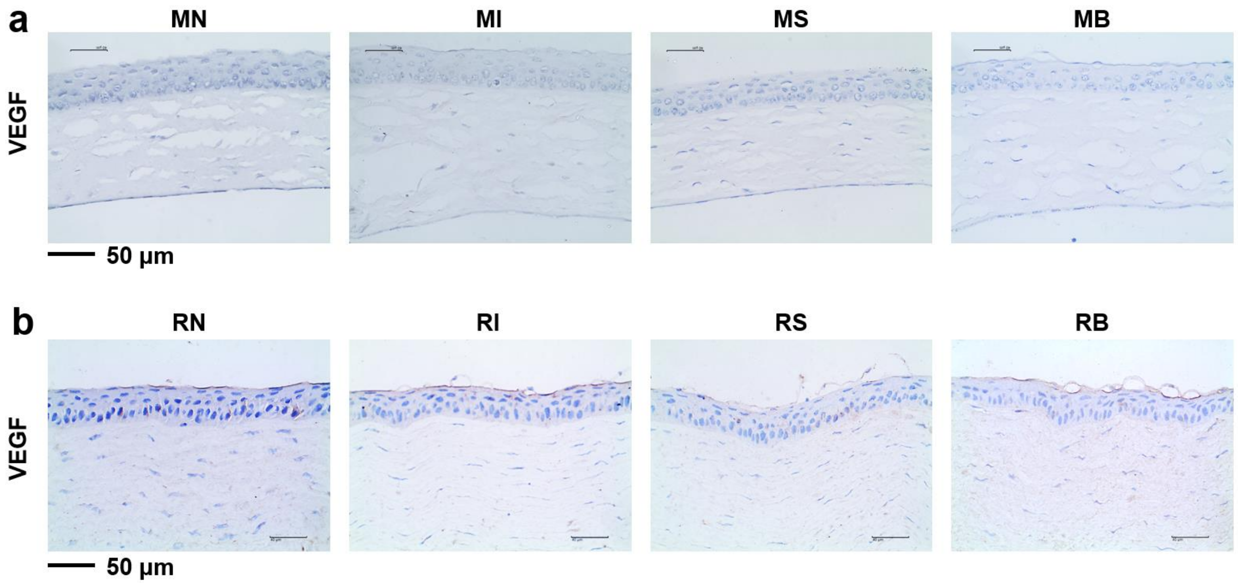

Supplementary Figure 23 | Mouse (a) and rabbit (b) IHC staining images of VEGF. Abbreviations: MN: normal mice without cornea injury; MI, mice in the intervention group; MS, mice in the sham group; MB, mice in the blank control group; RN: normal rabbits without cornea injury; RI, rabbits in the intervention group; RS, rabbits in the sham group; RB, rabbits in the blank control group; VEGF, vascular endothelial growth factor; IHC, immunohistochemistry.

Supplementary References

1. Gipson I. K. *et al.* Characteristics of a glycoprotein in the ocular surface glycocalyx. *Invest. Ophthalmol. Vis.* **33**, 218-227 (1992).
2. DelMonte, D. W. & Kim, T. Anatomy and physiology of the cornea. *Vision Res.* **37**, 588-598 (2011).
3. Schmucker, C. & Schaeffel, F. A paraxial schematic eye model for the growing C57BL/6 mouse. *Vision Res.* **44**, 1857-1867 (2004).
4. Bozkir, G. *et al.* Measurements of axial length and radius of corneal curvature in the rabbit eye. *Acta Med. Okayama* **51**, 9-11 (1997).
5. Macron, J., Gerratt, A. P. & Lacour, S. P. Thin hydrogel-elastomer multilayer encapsulation for soft electronics. *Adv. Mater. Technol.* **4**, 1900331 (2019).
6. Park, D. C. Carbonation of concrete in relation to CO_2 permeability and degradation of coatings. *Constr. Buildi. Mater.* **22**, 2260-2268 (2022).
7. Pye, D. G., Hoehn, H. H. & Panar, M. Measurement of gas permeability of polymers. I. Permeabilities in constant volume/variable pressure apparatus. *J. Appl. Polym. Sci.* **20**, 1921-1931 (1976).
8. Saccu, G. *et al.* Regenerative approaches and future trends for the treatment of corneal burn injuries. *J. Clin. Med.* **10**, 317 (2021).
9. Formisano, N. *et al.* Mechanical properties of bioengineered corneal stroma. *Adv. Healthc. Mater.* **10**, 2100972 (2021).
10. Dua, H. S., King, A. J. & Joseph, A. A new classification of ocular surface burns. *Br. J. Ophthalmol.* **85**, 1379 (2001).
11. Bai, J. Q., Qin, H. F. & Zhao, S. H. Research on mouse model of grade II corneal alkali burn. *Int. J. Ophthalmol.* **9**, 487-490 (2016).
12. Giacomini, C., Ferrari, G., Bignami, F. & Rama, P. Alkali burn versus suture-induced corneal neovascularization in C57BL/6 mice: An overview of two common animal models of corneal neovascularization. *Exp. Eye Res.* **121**, 1-4 (2014).
13. Yao, G. *et al.* Effective weight control via an implanted self-powered vagus nerve stimulation device. *Nat. Commun.* **9**, 5349 (2018).
14. Zhao, M. *et al.* Electrical signals control wound healing through phosphatidylinositol-3-OH kinase-γ and PTEN. *Nature* **442**, 457-460 (2006).
15. Song, B., Zhao, M., Forrester, J. V. & McCaig, C. D. Electrical cues regulate the orientation and frequency of cell division and the rate of wound healing *in vivo*. *Proc. Natl. Acad. Sci. USA* **99**, 13577-13582 (2002).
