# Supplementary material for: Snowflake-inspired and blink-driven flexible piezoelectric contact lenses for effective corneal injury repair
Source: Nat Commun. 2023 Jun 17;14:3604. doi: 10.1038/s41467-023-39315-6 (PMC10276863; doi:10.1038/s41467-023-39315-6)
Supplement: Supplementary file 2 — Description of additional supplementary files [file 41467_2023_39315_MOESM2_ESM.pdf]

Description of additional supplementary files

Supplementary Movie 1 : Finite element analysis of the BPCL attached to the eyeball.

Supplementary Movie 2 : Normal daily activity of a rabbit wearing the BPCL.

Supplementary Movie 3 : In vivo voltage output of the BPCL during human blink.

Supplementary Movie 4 : Voltage output stability of the BPCL.

Supplementary Movie 5 : Stability characterization under different deformation modes.
