# Supplementary material for: Snowflake-inspired and blink-driven flexible piezoelectric contact lenses for effective corneal injury repair
Source: Nat Commun. 2023 Jun 17;14:3604. doi: 10.1038/s41467-023-39315-6 (PMC10276863; doi:10.1038/s41467-023-39315-6)
Supplement: Supplementary file 8 — Reporting Summary [file 41467_2023_39315_MOESM8_ESM.pdf]

nature portfolio

Corresponding author(s): Yuan Lin

Last updated by author(s): Jun 3, 2023

Reporting Summary

Nature Portfolio wishes to improve the reproducibility of the work that we publish. This form provides structure for consistency and transparency in reporting. For further information on Nature Portfolio policies, see our Editorial Policies and the Editorial Policy Checklist.

Statistics

For all statistical analyses, confirm that the following items are present in the figure legend, table legend, main text, or Methods section.

n/a	Confirmed	
☐	☒	The exact sample size (*n*) for each experimental group/condition, given as a discrete number and unit of measurement
☐	☒	A statement on whether measurements were taken from distinct samples or whether the same sample was measured repeatedly
☐	☒	The statistical test(s) used AND whether they are one- or two-sided<br>*Only common tests should be described solely by name; describe more complex techniques in the Methods section.*
☒	☐	A description of all covariates tested
☒	☐	A description of any assumptions or corrections, such as tests of normality and adjustment for multiple comparisons
☐	☒	A full description of the statistical parameters including central tendency (e.g. means) or other basic estimates (e.g. regression coefficient) AND variation (e.g. standard deviation) or associated estimates of uncertainty (e.g. confidence intervals)
☐	☒	For null hypothesis testing, the test statistic (e.g. *F*, *t*, *r*) with confidence intervals, effect sizes, degrees of freedom and *P* value noted<br>*Give P values as exact values whenever suitable.*
☒	☐	For Bayesian analysis, information on the choice of priors and Markov chain Monte Carlo settings
☒	☐	For hierarchical and complex designs, identification of the appropriate level for tests and full reporting of outcomes
☒	☐	Estimates of effect sizes (e.g. Cohen's *d*, Pearson's *r*), indicating how they were calculated

Our web collection on statistics for biologists contains articles on many of the points above.

Software and code

Policy information about availability of computer code

Data collection	The electrical performance of all devices was measured by a Keithley 6514 electrometer and a portable DSO-X2012A oscilloscope. No commercial, open-source, nor custom code was used for data collection.
Data analysis	OriginPro 9, ABAQUS 6.12, and ImageJ V1.8.0. were used for data analysis, plotting, and statistics.

For manuscripts utilizing custom algorithms or software that are central to the research but not yet described in published literature, software must be made available to editors and reviewers. We strongly encourage code deposition in a community repository (e.g. GitHub). See the Nature Portfolio guidelines for submitting code & software for further information.

Data

Policy information about availability of data

All manuscripts must include a data availability statement. This statement should provide the following information, where applicable:

- Accession codes, unique identifiers, or web links for publicly available datasets
- A description of any restrictions on data availability
- For clinical datasets or third party data, please ensure that the statement adheres to our policy

The authors declare that all data supporting the findings of this study are available within the Article and its Supplementary Information. Source data are provided with this paper.

Human research participants

Policy information about studies involving human research participants and Sex and Gender in Research.

Reporting on sex and gender	Three healthy subjects participated in voltage monitoring, including two men (Participant I: 23 years old; Participant II: 26 years old) and one woman (24 years old).
Population characteristics	The voltage output in the human-worn state was monitored for proof-of-principle testing, and the non-invasive test did not involve tissue damage or biological characterization.
Recruitment	All volunteers participating in the study are entirely voluntary. The voltage output in the human-worn state was monitored for proof-of-principle testing, and the non-invasive test did not involve tissue damage or biological characterization. All volunteers are recruited within the school, stating the duration (30 minutes) and compensation amount ($50). This recruitment excludes individuals with low tolerance to wearing contact lenses. During the participation in the experiment, a principle verification test was conducted to monitor the voltage output of daily blinking while the volunteers were wearing it. Subjects have read the relevant research materials and received satisfactory answers to all questions. Subjects fully understand the relevant medical research materials and the potential risks and benefits of the research. The subjects know that participating in the study is voluntary and have the right to withdraw at any time. The subjects agree to the review of research materials by the drug regulatory department, ethics committee, or applicant and have expressed their willingness to participate in the study. All collected data and information are for research purposes only. The subjects agree to publish research results (including age, sex, and Supplementary Movie 3) in scientific journals or present at scientific conferences.
Ethics oversight	All experiments performed with animal and human participants were conducted under a standard protocol (1061420210617007) approved by the Ethics Committee of the Animal Experiment Center of University of Electronic Science and Technology of China.

Note that full information on the approval of the study protocol must also be provided in the manuscript.

Field-specific reporting

Please select the one below that is the best fit for your research. If you are not sure, read the appropriate sections before making your selection.

☒ Life sciences ☐ Behavioural & social sciences ☐ Ecological, evolutionary & environmental sciences

For a reference copy of the document with all sections, see nature.com/documents/nr-reporting-summary-flat.pdf

Life sciences study design

All studies must disclose on these points even when the disclosure is negative.

Sample size	Reid et al. made a circular lesion through the corneal epithelium using a trephine on Sprague Dawley rats (n = 4). In addition, they regulated corneal healing by controlling the electrical current at the edge of corneal injury in vivo (The FASEB Journal 19, 379-386, 2005).<br><br>Zhao et al. made a circular lesion through the whole corneal epithelium using a trephine on Sprague Dawley rats (from 3~12 independent eyes). They modulated corneal epithelial wound healing by controlling endogenous wound electric currents in vivo (Nature 540, 379, 2016).<br><br>KKanavi et al. established a corneal alkali burn model on New Zealand rabbits (n=10). They studied the repair effects of the pulsed electromagnetic field and pulsed low-level laser on the cornea (Experimental Eye Research 145, 216-223, 2016).<br><br>Wu et al. established a corneal epithelial wound model on the eyes of New Zealand rabbits (n = 3). They demonstrated that the electrical stimulation could accelerate the recovery of corneal wounds on rabbits' eyes in vivo (Advanced Science 2202506, 2022).<br><br>Our current sample size (n=10) is sufficient, exceeding most experiments related to corneal injury repair. Mice in the intervention (MI) group were intervened with an EF generated by the BPCL. Mice in the sham (MS) group were stimulated with deactivated devices in which the electrodes were disconnected from the BPCL. The mice in the blank (MB) control group had no wearable electrodes. Normal mice without cornea injury were labeled as MNn (n = 10, Male: n= 1-5, Female: n= 6-10). Mice in MI, MS, and MB groups were subjected to the same corneal injury surgery procedure, and the mice were labeled as MIn, MSn, and MBn (n = 10, Male: n= 1-5, Female: n= 6-10). Identical to the grouping of mice, the rabbits were divided into four groups (RN, RI, RS, and RB) and labeled as RNn, RIn, RSn, and RBn (n = 10, Male: n= 1-5, Female: n= 6-10). Both male and female animals were considered and employed to increase statistical robustness.
Data exclusions	No data was excluded.
Replication	The sample size of experimental animals (mice and rabbits) was 10 to ensure replicability and statistical robustness. From the measured results, the data showed high similarity within the same testing group, and the replicability was good in each group. Attempts at replication were successful. For representative experiments (Figs. 1c, h; 3b, h; 4f; Supplementary Figures 2d; 5b; 11c, d; 19c; 22a, b, c; 23a, b), each experiment was repeated independently many times (≥3) with similar results, demonstrating good data reproducibility.
Randomization	Mice and rabbits were randomly allocated into experimental groups. For mice and rabbits, males were labeled as 1-20, and females were

labeled as 21-40. Then, we randomly divided animals numbered 1-20 into four groups and 21-40 into four groups. Then randomly combine the grouped males and females. Mice in the sham (MS) group were stimulated with deactivated devices in which the electrodes were disconnected from the BPCL. The mice in the blank (MB) control group had no wearable electrodes. Normal mice without cornea injury were labeled as MNn (n = 10, Male: n= 1-5, Female: n= 6-10). Mice in MI, MS, and MB groups were subjected to the same corneal injury surgery procedure, and the mice were labeled as MIn, MSn, and MBn (n = 10, Male: n= 1-5, Female: n= 6-10). Identical to the grouping of mice, the rabbits were divided into four groups (RN, RI, RS, and RB) and labeled as RNn, RIn, RSn, and RBn (n = 10, Male: n= 1-5, Female: n= 6-10). Both male and female animals were considered and employed to increase statistical robustness.

Blinding

N/A. Only one condition that the devices work properly (BPCL intervention group) can affect the results in mouse and rabbit models. There were no significant differences in the other control groups (Sham and Blank). Therefore, the blinding process won't influence the result.

Reporting for specific materials, systems and methods

We require information from authors about some types of materials, experimental systems and methods used in many studies. Here, indicate whether each material, system or method listed is relevant to your study. If you are not sure if a list item applies to your research, read the appropriate section before selecting a response.

Materials & experimental systems

n/a	Involved in the study
☐	☒ Antibodies
☐	☒ Eukaryotic cell lines
☒	☐ Palaeontology and archaeology
☐	☒ Animals and other organisms
☒	☐ Clinical data
☒	☐ Dual use research of concern

Methods

n/a	Involved in the study
☒	☐ ChIP-seq
☒	☐ Flow cytometry
☒	☐ MRI-based neuroimaging

Antibodies

Antibodies used

Anti-TGF-β-APC (Biolegend, Clone TW7-16B4, Catalog: 141406, Dilution: 1:400, Mouse Antibody), Anti-α-SMA-antibody (Biolegend, Clone 1A4, Catalog: 614852, Dilution:1:500, Mouse Antibody), Anti-TGF-β-antibody (Abcam, ab215715, Dilution: 1:500, Rabbit Antibody), Anti-α-SMA-antibody (Abcam, ab5694, Dilution: 1:200, Rabbit Antibody), Anti-MMP9-antibody (Abcam, ab283575, Dilution: 1:500, Mouse/Rabbit Antibody), Anti-Keratin 3-antibody (Abcam, ab77869, Dilution: 1:200, Mouse/Rabbit Antibody), Anti-Keratin 12-antibody (Abcam, ab185627, Dilution: 1:1000, Mouse/Rabbit Antibody).

Validation

All antibodies were validated for the species and application used as stated in the product data sheet of each antibody provided by the manufacturer. All validation statements can be found on the respective antibody website:
1. Anti-mouse-TGF-β-APC: https://www.biolegend.com/en-us/products/apc-anti-mouse-lap-tgf-beta1-antibody-7310
2. Anti-mouse-α-SMA-antibody: https://www.biolegend.com/en-us/products/purified-anti-alpha-smooth-muscle-actin-antibody-23005
3. Anti-rabbit-TGF-β-antibody: https://www.abcam.cn/products/primary-antibodies/tgf-beta-1-antibody-epr21143-ab215715.html
4. Anti-rabbit-α-SMA-antibody: https://www.abcam.cn/products/primary-antibodies/alpha-smooth-muscle-actin-antibody-ab5694.html
5. Anti-mouse/rabbit-MMP9-antibody: https://www.abcam.cn/products/primary-antibodies/mmp9-antibody-rm1020-ab283575.html
6. Anti-mouse/rabbit-Keratin 3-antibody: https://www.abcam.cn/products/primary-antibodies/cytokeratin-3ck-3-antibody-ae5-ab77869.html
7. Anti-mouse/rabbit-Keratin 12-antibody: https://www.abcam.cn/products/primary-antibodies/keratin-12k12-antibody-epr17882-ab185627.html

Eukaryotic cell lines

Policy information about cell lines and Sex and Gender in Research

Cell line source(s)

Mouse fibroblast 3T3 cells (SCSP-5038) were obtained from the Cell Bank, Shanghai Institutes for Biological Sciences, Chinese Academy of Sciences.

Authentication

Identity of the cell lines were frequently checked by their morphological features but have not been authenticated by the short tandem repeat profiling.

Mycoplasma contamination

The cell line was tested for mycoplasma contamination. No mycoplasma contamination was found.

Commonly misidentified lines (See ICLAC register)

No commonly misidentified cell lines are used in this study.

Animals and other research organisms

Policy information about studies involving animals; ARRIVE guidelines recommended for reporting animal research, and Sex and Gender in Research

Laboratory animals	Eight-week-old C57BL/6 mice and eight-week-old New Zealand white rabbits were acquired from Dashuo Laboratory Animal Co., Ltd (Chengdu, China). All animals were housed in separated cages in a temperature-controlled (22°C) room (relative humidity: 45%~60%) with a 12-hour light/12-hour dark cycle with free water and feed access. All animal-based procedures were performed under the National Institutes of Health guidelines for the care and use of laboratory animals.
Wild animals	This study did not involve wild animals.
Reporting on sex	Eight-week-old C57BL/6 mice and eight-week-old New Zealand white rabbits were subjected to the same corneal injury procedure and fed under the same conditions. The mice were divided into four groups and labeled as MNn, MIn, MSn, and MBn (n = 10, Male: n= 1-5, Female: n= 6-10; Both male and female mice were considered and employed to increase statistical robustness.). Identical to the grouping of mice, the rabbits were labeled as RNn, RIn, RSn, and RBn (n = 10, Male: n= 1-5, Female: n= 6-10; Both male and female rabbits were considered and employed to increase statistical robustness.).
Field-collected samples	No field collected samples were used in the study.
Ethics oversight	All experiments performed with animal and human participants were conducted under a standard protocol (1061420210617007) approved by the Ethics Committee of the Animal Experiment Center of University of Electronic Science and Technology of China.

Note that full information on the approval of the study protocol must also be provided in the manuscript.
